# Multimodal EEG–MRI Neuroimaging in Schizophrenia—A Systematic and Mechanistic Review

**DOI:** 10.3390/jcm15114306

**Published:** 2026-06-02

**Authors:** James Chmiel, Marta Kopańska

**Affiliations:** 1 Institute of Physical Culture Sciences, Faculty of Physical Culture and Health, University of Szczecin, Al. Piastów 40B Block 6, 71-065 Szczecin, Poland; 2 Department of Medical Communication and Professional Competency Development, Faculty of Medicine, Collegium Medicum, University of Rzeszów, 35-310 Rzeszów, Poland; mkopanska@ur.edu.pl

**Keywords:** schizophrenia, neuroimaging, EEG, fMRI, multimodal, neurophysiology

## Abstract

**Introduction**: Schizophrenia is characterised by distributed abnormalities in electrophysiological dynamics and large-scale brain networks, yet unimodal EEG or MRI alone cannot fully explain how fast neural computations relate to spatially organised circuit dysfunction. Multimodal EEG–MRI approaches offer a bridge across temporal and anatomical scales by explicitly modelling cross-modal coupling. **Methods**: Following PRISMA 2020 guidance, we conducted a systematic, mechanistic review of human studies (adults ≥ 18 years) comparing schizophrenia-spectrum groups with healthy controls using EEG combined with at least one MRI modality (fMRI, structural MRI, and/or diffusion MRI) and explicit EEG–MRI integration (e.g., EEG-informed fMRI, joint ICA, mCCA/MCCA, coupled matrix–tensor factorisation, DCM-based fusion). Searches were performed in PubMed/MEDLINE, Embase, Web of Science, Scopus, PsycINFO, IEEE Xplore, ResearchGate, and Google Scholar for January 2000–December 2025, supplemented by citation tracking. Risk of bias was assessed with ROBINS-I, and due to heterogeneity, results were synthesised narratively by integration of families. **Results**: From 148 records, 23 studies met the inclusion criteria. Studies used mainly simultaneous EEG–fMRI at 3T and spanned resting-state designs and task paradigms dominated by auditory processing (oddball, MMN/N100–P200, ASSR/aeGBR), with additional work in affective context, working memory, semantic processing (N400), sensory gating, and pharmacologic challenge. Across tasks, the most reproducible multimodal signature was disrupted coupling between electrophysiological markers and the recruitment of large-scale networks, rather than isolated changes in EEG or fMRI metrics. Target detection/oddball paradigms converged on reduced late ERP responses (especially P300, sometimes N2) alongside reduced expression or loss of coupling to salience/ventral attention and control circuitry (including ACC/anterior insula/TPJ). Resting-state studies most consistently indicated altered “coupling rules” (frequency specificity, timing/lag structure, and directionality), including abnormalities detectable even when unimodal summaries were weak. Extended multimodal studies (adding sMRI/DTI and/or classification) suggested that combining modalities can improve discrimination, though performance was sensitive to sample size, demographic imbalance, and feature-selection/validation choices. **Conclusions**: Multimodal EEG–MRI studies support schizophrenia as a disorder involving persistent structural and circuit-level abnormalities whose functional expression varies dynamically across cognitive states and task demands. Future progress will depend on harmonised acquisition/artefact-control practices for simultaneous EEG–fMRI, larger and more diverse samples (including early/CHR and longitudinal designs), and cross-site replication of mechanistically interpretable coupling biomarkers.

## 1. Introduction

Schizophrenia is a severe, heterogeneous disorder characterised by psychotic symptoms, cognitive impairment, and substantial functional disability. Converging evidence supports schizophrenia as a brain-based illness with distributed abnormalities spanning microcircuit physiology, large-scale network dynamics, and neuroanatomy. Among available tools, electroencephalography (EEG) and magnetic resonance imaging (MRI) are the two most widely used noninvasive neuroimaging approaches in schizophrenia research, and, importantly, they interrogate complementary levels of organisation: EEG provides a direct, millisecond-scale window into neural population dynamics. At the same time, MRI offers millimetre-scale characterisation of brain structure and systems-level connectivity. Reviews across several decades show that schizophrenia is associated with reproducible—though individually variable—alterations in both electrophysiological markers and MRI-derived brain phenotypes, motivating integrative multimodal work that can relate where abnormalities occur to when and how neural computations break down.

### 1.1. EEG Findings in Schizophrenia

EEG abnormalities in schizophrenia have often been framed as disturbances of information processing and neural coordination, measurable as changes in event-related potentials (ERPs), oscillatory activity, and functional coupling. One of the most robust ERP findings concerns early auditory prediction and salience signalling; mismatch negativity (MMN) is reliably reduced in schizophrenia, with meta-analytic syntheses indicating consistent impairment and relevance to illness stage and functional outcome. These MMN abnormalities are frequently interpreted within predictive-processing accounts, in which impaired formation or updating of sensory expectations contributes to downstream cognitive and clinical symptoms [1].

A second well-replicated ERP signature involves later, attention- and context-updating processes. P300 amplitude reductions and latency alterations have been documented for decades and consolidated in both classic and contemporary meta-analytic/review treatments, which emphasise trait-like features alongside modulation by symptoms, task demands, and illness course. Earlier sensory-gating and perceptual components are also frequently implicated [2]. Reviews of P50 sensory gating describe diminished suppression to repeated auditory stimuli in a substantial proportion of patients, often discussed as reflecting inhibitory filtering deficits linked to hippocampal and cholinergic mechanisms [3]. Likewise, reviews focusing on the auditory N100 component report reduced amplitudes in schizophrenia and consider its potential relationship to vulnerability markers and perceptual dysfunction [4]. At higher levels of cognition, ERP-focused reviews of the N400 describe atypical semantic/context processing and impaired use of predictive meaning constraints—findings that align with broader cognitive and language disturbances observed clinically [5]. Importantly, these ERP abnormalities are not merely “task effects”; meta-analytic reviews addressing multiple auditory ERP endophenotypes highlight that several components (e.g., P50, MMN, P300) show measurable alterations across patient samples and, in some cases, in relatives or high-risk groups, supporting their use in mechanistic and translational models [6,7].

Beyond time-locked ERPs, reviews of schizophrenia EEG increasingly emphasise abnormalities of neural oscillations and synchrony—candidate mechanisms for impaired perceptual binding, cognitive control, and working memory [8,9,10]. Influential syntheses argue that disturbed coordination in beta- and gamma-band rhythms is a central feature of schizophrenia, potentially reflecting disruptions in excitation–inhibition balance and interneuron-mediated rhythm generation [8,11,12]. Complementing these conceptual accounts, reviews focusing on gamma activity more broadly link abnormal γ-band dynamics to perceptual and cognitive deficits and to GABAergic/parvalbumin-interneuron dysfunction in both clinical and preclinical literatures [9,11]. A particularly well-studied oscillatory probe is the 40 Hz auditory steady-state response (ASSR); meta-analytic evidence indicates robust reductions in 40 Hz ASSR power and phase locking in schizophrenia, and newer reviews discuss its utility as a circuit-level biomarker sensitive to mechanisms of cortical synchronisation [13,14].

Resting-state EEG work, as summarised in reviews, further suggests that schizophrenia is associated with shifts in spectral power and coordination—often described as increases in slower activity and/or alterations in alpha rhythms—though patterns can vary with recording conditions, chronicity, and medication exposure [15,16]. Finally, emerging review-level syntheses highlight abnormalities in EEG-derived microstate dynamics across psychosis-spectrum disorders, suggesting that brief, recurring scalp topographies (putatively reflecting large-scale network states) may index altered brain-state switching and salience processing in schizophrenia [17,18].

### 1.2. MRI Findings in Schizophrenia

MRI studies and reviews converge on schizophrenia as a disorder with widespread but regionally patterned differences in brain structure and connectivity. Early and classic quantitative syntheses established that schizophrenia is associated with regional volume reductions and ventricular enlargement, with consistent involvement of frontal and temporal regions and subcortical structures [19,20]. Voxel-based morphometry (VBM) meta-analyses further consolidated evidence for distributed grey matter deficits, particularly across prefrontal, superior temporal, and limbic areas, while also noting substantial heterogeneity attributable to clinical and methodological factors [21,22]. Reviews concentrating on first-episode cohorts indicate that many abnormalities are already present early in the illness, supporting neurodevelopmental contributions while leaving open questions about later progression [23].

Large-scale consortia and modern meta-analyses have refined these anatomical profiles with greater precision and statistical power. ENIGMA-based analyses report widespread cortical thinning (notably in frontal and temporal regions) and smaller cortical surface area in schizophrenia compared with controls [24]. Complementary ENIGMA work on subcortical volumes demonstrates a reproducible pattern involving structures such as the hippocampus, amygdala, thalamus, and basal ganglia—findings frequently discussed in relation to memory, salience, and thalamocortical integration [25]. More general neuroimaging overviews integrate these structural results with evidence for altered gyrification and accelerated grey matter loss in at least some patient subgroups, emphasising that effect sizes are typically modest-to-moderate at the group level but robust across large samples. Longitudinal meta-analytic work also supports the presence of progressive structural changes over time in schizophrenia, affecting both grey and white matter, while continuing to debate the relative roles of illness processes, treatment effects, and cohort differences [26,27].

White matter abnormalities are another central MRI theme. Diffusion MRI reviews and meta-analyses indicate reduced fractional anisotropy and altered diffusivity across multiple tracts, consistent with disrupted structural connectivity [28]. ENIGMA-DTI results provide a remarkably reproducible global profile of widespread white matter microstructural differences in schizophrenia across international cohorts [29]. At the network level, MRI-based connectivity reviews synthesise evidence that schizophrenia involves dysconnectivity across large-scale systems, including complex patterns in functional connectivity that can include both reductions and increases depending on network, context, and analytic approach [30].

### 1.3. Multimodal Neuroimaging EEG–MRI

The rationale for simultaneous EEG–fMRI emerged in the 1990s from both clinical demand and fundamental neuroscience, after early studies established that EEG could be recorded safely inside an MR scanner [31]. Initial uptake was driven mainly by epilepsy, where interictal discharges captured on EEG could be linked to spike-timed BOLD changes to help localise epileptogenic networks for presurgical evaluation [32,33]. From the early 2000s onward, the approach expanded to studies of normal cognition and perception [34,35,36,37]. Conceptually, EEG–fMRI leverages complementarity; EEG reflects fast electrophysiological activity (summated postsynaptic potentials), while fMRI indexes slower hemodynamic changes, enabling empirical mapping between neural events/oscillations and BOLD responses despite an incomplete understanding of neurovascular coupling.

Technically, concurrent acquisition is challenging because the MR environment induces significant EEG artefacts and imposes strict safety constraints [38,39]. Safety protocols and MR-compatible hardware (e.g., non-ferromagnetic components, careful lead layouts, resistive wiring) were established early and refined over time [40,41,42,43,44]. The dominant EEG contaminants are the gradient artefact and ballistocardiogram/pulse artefact [45,46]. Because gradient artefacts are periodic and time-locked to scanning, average artefact subtraction (AAS) became a foundational solution [45]. Subsequent work showed that combining template approaches with ICA-based cleaning often yields better performance than any single method alone [47,48,49], and that reference-sensor strategies (e.g., pickup coils/carbon-wire loops; reference-layer methods) can further improve artefact removal, especially for pulse-related artefacts [50,51,52]. Comparative evaluations generally support hybrid pipelines (e.g., AAS + ICA and/or reference sensors) as providing the most reliable EEG quality in-scanner [49,50,53].

EEG hardware can also affect fMRI, as electrodes and leads may introduce susceptibility-related dropout or local BOLD distortions, particularly at higher field strengths [54,55,56,57,58,59]. These effects are typically mitigated through low-susceptibility materials and careful cable management (e.g., minimising loops), and are generally considered manageable with modern systems [40,54,60,61,62]. As a result, simultaneous EEG–fMRI has progressed from proof-of-concept to a mature technique supported by established safety practices, dedicated hardware, and robust signal-processing methods.

With acquisition challenges addressed, a central question becomes how to integrate the modalities. Common asymmetrical strategies include EEG-informed fMRI, where EEG-derived events or continuous features are entered as regressors in fMRI models to identify BOLD correlates [36,63,64,65], and fMRI-constrained EEG, where fMRI activations/networks provide priors that regularise the EEG inverse problem and improve spatial specificity [66,67,68,69,70]. Model-based integration connects neural and hemodynamic signals using coupled generative frameworks (e.g., neural mass models with hemodynamic models such as Balloon-type formulations) to test hypotheses about neurovascular coupling and network interactions [71,72,73,74,75,76,77,78,79,80,81]. More recently, machine learning approaches have combined EEG and fMRI features for decoding/classification [82] and explored cross-modal prediction (e.g., learning mappings from EEG to fMRI-like representations) [83,84,85]. Collectively, these methods aim to link millisecond-scale electrophysiological dynamics to spatially distributed BOLD networks, offering a principled route to bridge mechanisms across scales in neuropsychiatric and neurological disorders.

The complementarity of EEG and fMRI is not merely technical but mechanistic. EEG provides direct, millisecond-scale sensitivity to neuronal population dynamics, including event-related potentials, oscillatory synchronisation, phase resetting, and transient changes in cortical excitability, but it has limited spatial specificity, especially for deep or spatially distributed generators [86,87,88]. In contrast, fMRI provides millimetre-scale localisation of task-related and resting-state BOLD activity across cortical, subcortical, cerebellar, and large-scale network systems, but the BOLD signal is temporally delayed and reflects neurovascular and metabolic consequences of neural activity rather than fast electrical signalling itself [89,90,91]. Therefore, combining EEG and fMRI allows the investigator to ask a question that neither modality can answer alone: which anatomically defined brain regions and networks are recruited at the precise moments when electrophysiological computations occur [92,93,94]? In practice, this means that EEG-derived markers such as MMN, N100/P200, P300, N400, gamma-band activity, alpha fluctuations, or phase synchronisation can be used to explain trial-by-trial or state-dependent BOLD variability, while fMRI-defined regions or networks can constrain the interpretation of EEG generators and connectivity [87,88,90,94]. This integrated approach can reveal whether an abnormal ERP or oscillatory response in schizophrenia reflects reduced recruitment of sensory cortex, impaired engagement of salience/control networks, altered thalamo–cortical coupling, disrupted default-mode suppression, or abnormal timing of neurovascular coupling [86,87,91,95]. Thus, multimodal EEG–fMRI moves beyond parallel description of “electrical” and “hemodynamic” abnormalities and instead tests cross-scale coupling between fast neural computations and spatially organised brain systems [89,92,93,94]. This is especially important in schizophrenia, where the disorder is unlikely to be explained by isolated abnormalities in either scalp electrophysiology or regional BOLD activation alone, but rather by disturbed coordination between microcircuit-level synchronisation, cognitive timing, and large-scale network recruitment [86,87,88,91]. Evidence from simultaneous EEG–fMRI and related integration studies shows that this approach can identify EEG–BOLD coupling, single-trial ERP–BOLD relationships, oscillation-linked resting-state networks, and multimodal components that are not visible, or are less interpretable, when EEG and fMRI are analysed separately [90,92,93,94,95].

As noted earlier, review-level evidence portrays schizophrenia as a condition with disrupted electrophysiological signatures of sensory prediction, salience allocation, and cognitive updating (e.g., MMN, P300), altered oscillatory synchronisation and circuit-level rhythmicity (gamma/beta, ASSR), and MRI-visible differences in cortical/subcortical anatomy, white matter organisation, and large-scale network connectivity. Yet EEG and MRI each provide an incomplete explanation on their own; EEG is highly sensitive to timing and dynamics but limited in spatial specificity, whereas MRI offers anatomical and systems-level localisation but only indirect access to fast neural computations. This complementarity motivates multimodal EEG–MRI neuroimaging as a strategy to bridge scales—from microcircuit synchronisation to macroscale network architecture—while addressing the core challenge emphasised across reviews; schizophrenia’s neurobiology is distributed, heterogeneous, and likely mechanistically plural, requiring approaches that can link structure, function, and dynamics within the same individuals and across illness stage of this systematic and mechanistic review. The aim of this review was to synthesise and analyse studies using multimodal EEG–MRI in schizophrenia. The review analysed patient characteristics, measurement methods, and EEG–MRI findings across studies and explored common patterns, differences, and potential neural mechanisms underlying multimodal neuroimaging findings.

## 2. Materials and Methods

### 2.1. Design, Reporting Framework, and Protocol

This systematic review was conducted according to PRISMA 2020 reporting guidance. The review question was defined a priori using a PICO-style framework targeting schizophrenia-spectrum populations studied with multimodal EEG–MRI neuroimaging (simultaneous or separate-session acquisition) and explicit cross-modal integration (e.g., EEG-informed fMRI, joint decomposition/fusion, multimodal connectivity modelling). The review was registered in the PROSPERO database (CRD420261396485).

### 2.2. Eligibility Criteria

Studies were included if they met all criteria below:

Population

1. Adults (≥18 years) with schizophrenia-spectrum psychotic disorders (schizophrenia, schizoaffective disorder, psychotic-spectrum disorders, or clinical high risk for psychosis if analysed as a psychosis-risk group) diagnosed using standardised clinical criteria (e.g., DSM/ICD; structured interviews such as SCID, MINI, SCAN).
2. A healthy control group was required for group-comparison aims; studies without controls were excluded.

Multimodal neuroimaging

1. EEG combined with at least one MRI modality: fMRI (task or resting-state), structural MRI, and/or diffusion MRI.
2. Acquisition could be simultaneous EEG–fMRI or separate sessions. Still, studies had to report a principled method for relating EEG and MRI measures across subjects/trials (not merely reporting both modalities independently).

Integration method (required)

1. Explicit multimodal integration, including but not limited to: EEG-informed fMRI (parametric modulation), joint ICA (jICA), multimodal CCA/MCCA, coupled matrix–tensor factorisation (e.g., ACMTF), fMRI-informed EEG source/DCM, cross-modal covariance mapping, or other model-based fusion linking EEG features to MRI-derived measures.

Outcomes

- At least one schizophrenia-related between-group difference or association with symptoms/cognition involving a multimodal metric (e.g., EEG–BOLD coupling, joint component loadings, multimodal effective connectivity, classification performance from multimodal features).

Study type and language

- Peer-reviewed original human research (cross-sectional, case–control, cohort, experimental task, or resting-state; pharmacologic challenge studies were eligible if multimodal integration was performed).
- Conference abstracts without full methods/results, animal studies, and purely methodological papers without patient data were excluded.
- Language restrictions: Only English-language publications were included due to feasibility constraints and because the vast majority of multimodal EEG–MRI schizophrenia studies indexed in major biomedical databases are published in English. Nevertheless, this restriction may have introduced language bias and potentially excluded relevant regional literature.

### 2.3. Information Sources

Electronic searches were performed in the following databases: PubMed/MEDLINE, Embase, Web of Science, Scopus, PsycINFO, IEEE Xplore, Research Gate, and Google Scholar from January 2000 to December 2025. In addition, reference lists of included papers and relevant reviews were hand-searched. Forward citation tracking was performed.

### 2.4. Search Strategy

Search strings combined terms for (i) schizophrenia/psychosis, (ii) EEG, (iii) MRI/fMRI/DTI/structural MRI, and (iv) multimodal fusion/integration. A representative strategy was:

- (“schizophrenia” OR “schizoaffective” OR psychosis OR “psychotic disorder” OR “clinical high risk” OR prodrom*) AND (EEG OR electroencephalogra* OR ERP OR oscillation* OR “event-related”) AND (fMRI OR “functional MRI” OR MRI OR “structural MRI” OR diffusion OR DTI) AND (“simultaneous” OR “EEG-informed” OR “data fusion” OR “joint ICA” OR jICA OR “canonical correlation” OR mCCA OR MCCA OR “coupled matrix tensor” OR ACMTF OR “dynamic causal modeling” OR DCM OR “covariance mapping”).

### 2.5. Study Selection Process

Records were de-duplicated and screened in two stages:

1. Title/abstract screening by two independent reviewers.
2. Full-text screening by two independent reviewers against eligibility criteria.

**Figure 1:**
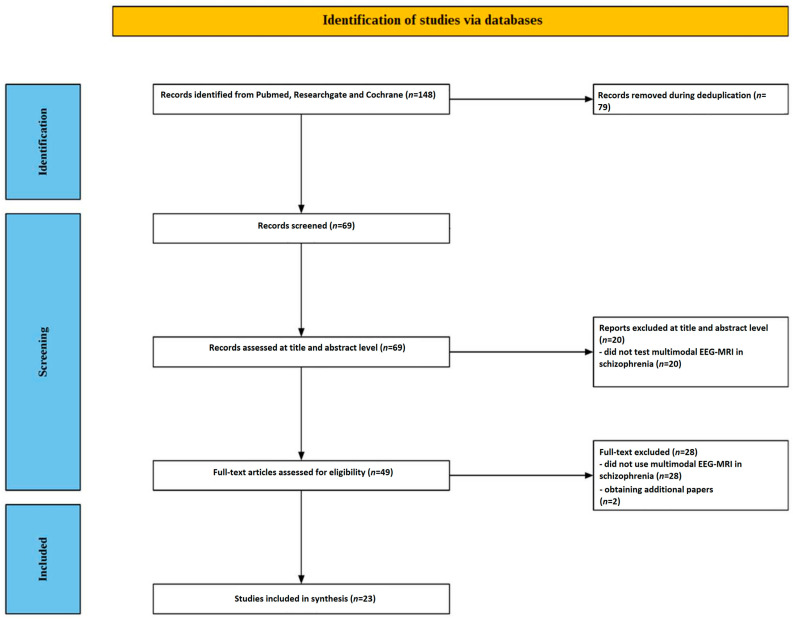
Flow chart depicting the different phases of the systematic review.

Disagreements were resolved by consensus; if unresolved, a third reviewer adjudicated. Reasons for exclusion at the full-text stage were recorded. The selection process is summarised using a PRISMA flow diagram (Figure 1). The PRISMA checklist is presented in Supplementary Material.

### 2.6. Data Extraction and Management

A standardised extraction form was piloted on a subset of studies and then applied to all included studies by two independent reviewers. Extracted data were cross-checked; discrepancies were reconciled by consensus.

Extracted domains

- Study metadata: author, year, country/site(s), design (task/rest; simultaneous vs. separate sessions), sample size analysed, exclusions (e.g., motion, unusable EEG decomposition).
- Participants: diagnostic criteria and instruments, illness duration, symptom scales (PANSS, SAPS/SANS, BPRS, SOPS), cognitive measures, demographics (age/sex), handedness, smoking status if available.
- Medication and clinical context: antipsychotic exposure (chlorpromazine/risperidone/olanzapine equivalents), adjunct medications, medication-free status where reported.
- Paradigm: resting state (eyes open/closed), auditory/visual oddball, working memory, affective/face processing, somatosensory gating, etc.
- Acquisition parameters: scanner field strength, fMRI TR/TE, EEG channels/sampling rate, synchronisation approach, sparse sampling (if used), and artefact correction methods (gradient/BCG correction, ICA/CCA/FOOOF, etc.).
- Multimodal integration method: EEG-informed GLM/PPI, joint decomposition (jICA/mCCA/MCCA), coupled factorisation (ACMTF), model-based connectivity (EEG–DCM, spectral DCM), cross-modal lag/covariance mapping, classification pipelines.
- Primary multimodal outcomes: direction/magnitude of group effects, networks/regions implicated, effective/functional connectivity changes, joint component differences, coupling timing/lag effects, classification metrics (accuracy/sensitivity/specificity/AUC) where relevant.
- Secondary outcomes: relationships with symptoms (e.g., PANSS dimensions, avolition/apathy, sensory gating), stress, illness duration, age of onset, and medication dose.

### 2.7. Outcome Definitions (Review-Level)

Given heterogeneity across paradigms and fusion methods, outcomes were pre-specified into the following families:

1. EEG–BOLD coupling outcomes
  - Parametric EEG-informed fMRI effects (trial-to-trial EEG features predicting BOLD).
  - Resting coupling measures (e.g., EEG power/synchronisation metrics linked to BOLD fluctuations), including lag/timing analyses.
2. Joint/fused component outcomes
  - Group differences in subject loadings of joint components (e.g., jICA, mCCA/MCCA, ACMTF), with associated EEG waveforms and MRI spatial maps.
3. Multimodal connectivity and mechanistic model outcomes

Effective connectivity parameters (e.g., DCM-derived top-down/backward vs. bottom-up/forward influences), and relationships to symptoms.

4. Prediction/classification outcomes (if present)
  - Diagnostic discrimination using multimodal features (e.g., accuracy, sensitivity/specificity, cross-validation design, held-out testing).

### 2.8. Risk of Bias and Quality Assessment

Because included studies span observational case–control designs, task experiments, and machine-learning classification pipelines, risk of bias was assessed using a ROBINS-I. Each study was rated independently by two reviewers; disagreements were resolved by consensus. Results are summarised in a risk of bias in Table 2.

Risk of bias was assessed independently by two reviewers using the ROBINS-I framework, which evaluates bias across multiple domains including confounding, participant selection, classification of interventions/exposures, missing data, outcome measurement, and selective reporting. Disagreements between reviewers were resolved through consensus discussion; where necessary, a third reviewer adjudicated unresolved discrepancies. Studies judged to have more substantial methodological limitations or higher risk of bias were interpreted more cautiously during narrative synthesis, particularly when findings lacked replication across independent cohorts or paradigms.

### 2.9. Data Synthesis and Effect Measures

Given expected heterogeneity (tasks, EEG features, MRI targets, fusion models, and statistical thresholds), the primary synthesis was structured narrative synthesis grouped by integration family.

## 3. Results

Figure 1 provides a summary of the screening process. Of the 148 studies initially identified through database search, 79 duplicates were removed. Of the remaining 69 publications, 20 were removed based on title and abstract review. Forty-nine articles were submitted for full-text review. At this stage, 28 articles were excluded because they did not use multimodal EEG–MRI neuroimaging in schizophrenia. The remaining 21 studies that fit the scope of the review were searched for cited, citing, and similar articles. This resulted in two additional matching publications. Ultimately, 23 studies were included in the review [96,97,98,99,100,101,102,103,104,105,106,107,108,109,110,111,112,113,114,115,116,117,118]. Table 1 provides a summary of included studies.

### 3.1. Participant Characteristics

Across the included multimodal EEG–MRI studies, participant samples spanned small, highly controlled cohorts to large case–control datasets. Most investigations contrasted individuals with schizophrenia-spectrum illness against healthy controls. However, several studies broadened the clinical spectrum to include schizoaffective disorder, brief/acute psychotic disorders, clinical high-risk (CHR/HRP) states, and (in one extensive mechanistic modelling study) first-degree relatives.

#### 3.1.1. Sample Composition and Size

Study sizes varied markedly, with schizophrenia-spectrum patient samples ranging from *n* = 11 to *n* ≈ 107 (e.g., [99,108,109]) and control groups of comparable size. Several studies analysed psychosis-spectrum cohorts rather than “schizophrenia only,” combining schizophrenia and schizoaffective disorder [98,101,107] or including acute/brief psychotic disorder alongside schizophrenia [105,107,109]. One study focused on clinical high risk for psychosis (HRP; *n* = 24) compared with matched controls (*n* = 24) [106]. Another included first-degree relatives (*n* = 57) in addition to schizophrenia and control groups, with usable sample sizes varying by paradigm after quality control [104]. Two-site data pooling was also employed in a resting EEG–fMRI coupling study (SFVAMC and Bern), yielding 42 psychotic patients and 37 controls [107].

#### 3.1.2. Diagnosis and Diagnostic Verification

Clinical diagnoses were typically established with structured interviews and contemporary diagnostic systems: SCID-based DSM-IV/DSM-5 procedures were standard (Ref. [96] used SCID-I verification; Refs. [98,99,100,104,108,111,116,117] used SCID variants), while ICD-10 criteria were used or co-reported in several European cohorts [96,105,107,109]. One male-only schizophrenia cohort was diagnosed with SCAN [100]. HRP status in the early-detection study was defined via German Research Network on Schizophrenia (GNRS) criteria, with MINI used to rule out schizophrenia-spectrum disorders and characterise comorbidity [11]. Control participants were uniformly screened to exclude psychiatric illness and—frequently—first-degree family history of psychosis [96,98,99,101,102,106,107,109,114,115,116,117].

#### 3.1.3. Demographic Characteristics

Most studies recruited adults spanning late adolescence through older adulthood, with explicit eligibility windows commonly set at 18–55 years (e.g., [96,111]), but realised samples sometimes extended to the late 60s [101]. Mean ages across cohorts generally clustered in the 30s to mid-40s (e.g., [96,101,107,108,113,114,116]), with HRP participants being younger (early 20s; [106])—sex distributions varied by sample and recruitment setting. Many schizophrenia cohorts were male-predominant (e.g., VA-based studies [107,116]; several task EEG–fMRI datasets [101,102]), but there were notable exceptions: One large resting EEG–fMRI dataset from China was predominantly female in both patient and control groups [100]. One sensory-gating study included men only [110]. Handedness criteria were often restrictive (frequently right-handed only, e.g., [100,105,109,113]), though some studies included a small number of left-handed participants [97,102]. Group matching on age and sex was standard (e.g., [96,98,105,107,109,114]), but not universal; where mismatches existed (e.g., age/sex differences in [97] and age differences in [100]), analyses typically regressed or covaried demographic factors.

#### 3.1.4. Clinical Characteristics, Chronicity, and Symptom Severity

Illness chronicity ranged from early/shorter-duration samples to chronic outpatient cohorts. Reported mean illness duration often fell in the ~6–14 year range (e.g., [96,102,109,113,115]), with some larger cohorts reporting means around a decade or more [100,108]. Symptom severity was most commonly quantified using the PANSS [96,100,101,102,105,107,108,109,111,113,115,117], with many samples characterised as mild-to-moderately symptomatic or clinically stable outpatients (e.g., [96,111,116]). Other symptom instruments included SAPS/SANS (including avolition/apathy emphasis; [98]), BPRS positive/negative scales [104] or UCLA-BPRS [116], SSPI [112], and prodromal ratings via SOPS in HRP [106]. Several studies also reported cognition/functional measures (e.g., neuropsychological tests, IQ estimates, depression/alexithymia, and functioning in [96]; education differences addressed as covariates in [102,107]).

#### 3.1.5. Medication Status and Special Exposure Groups

Most schizophrenia-spectrum participants were medicated, often predominantly with atypical antipsychotics [96,109,112,114,116]. Medication dose was frequently summarised as chlorpromazine equivalents [100,105,107,108,109,111,115,116] or risperidone equivalents [102,113]. Some studies included mixed medication status; a subset of patients were not taking antipsychotics in an auditory EEG–fMRI fusion study [98], and in the male-only gating cohort, six were medication-free (no antipsychotics in the prior three months) while others received typical/atypical agents including clozapine [110]. One study focused specifically on smokers and acute nicotine administration in a crossover design, excluding clozapine users due to potential EEG effects [111]. HRP participants had limited medication exposure (a small subset on low-dose atypical antipsychotics and/or antidepressants; [106]).

#### 3.1.6. General Inclusion/Exclusion Criteria and Quality Control

Across studies, standard exclusions included major neurological illness, significant head injury, MRI contraindications, and recent substance abuse/dependence (often stricter for controls than patients; [98,101,107,115,117]). Sensory prerequisites were task-dependent (e.g., normal/corrected vision [96], adequate hearing thresholds [106], normal hearing self-report/practice success [99,114]). Quality-control attrition was nontrivial in simultaneous EEG–fMRI settings, with exclusions due to excessive motion (e.g., [100,110,111]) and unusable EEG decompositions or poor EEG quality (e.g., [98,101,106]). Some paradigms had additional signal-specific attrition; notably, the somatosensory P50 gating study excluded participants lacking an identifiable P50 waveform, yielding reduced EEG analysable subsamples [110].

### 3.2. Paradigms Used

Across the reviewed multimodal EEG–MRI studies, paradigms concentrated around resting-state acquisitions and a set of well-established task families—most prominently auditory paradigms—chosen to elicit canonical electrophysiological markers (e.g., MMN, N100/P200, N2/P3/P300, N400, aeGBR, ASSR) while providing fMRI access to the spatial organisation of the underlying networks. A substantial subset of studies used simultaneous resting-state EEG–fMRI to characterise intrinsic coupling between ongoing electrophysiology and BOLD activity or connectivity. Resting scans were implemented with either eyes-open fixation ([101,115], and the harmonised eyes-open condition emphasised in the two-site cohort in [107]) or eyes-closed instructions to relax while avoiding sleep [100,109,113]. Rest was not always treated as a neutral baseline; one study explicitly motivated the “resting” scan as a structured sensory context because of scanner acoustics and tested how moment-to-moment EEG fluctuations map onto BOLD in auditory cortex and beyond [101]. Resting-state paradigms supported diverse EEG features and coupling models, including residual/band-limited power after accounting for the aperiodic spectrum (e.g., residual alpha; [115]), low-gamma power and 1/f exponent estimates [101], global phase synchronisation (GFS; [107]), dynamic directed rhythm information used as a modulator of thalamo–cortical and triple-network connectivity [100], and traveling-wave directionality (forward vs. backward propagation) paired with fMRI-derived cortical hierarchy/gradient organization [108]. Some resting studies additionally evaluated connectivity structure more directly (e.g., DMN-focused fMRI connectivity paired with EEG source-space lagged coherence; [113]).

Task-based paradigms were dominated by audition, reflecting both the robustness of auditory EEG biomarkers in schizophrenia research and the feasibility of eliciting reliable responses during MRI. The auditory oddball family appeared repeatedly, typically contrasting frequent standard tones with infrequent targets requiring a button press and, in some variants, infrequent novel sounds. These designs were used to probe attentional orienting and salience detection and to link ERP components such as N2 and P3/P300 to fMRI activation patterns via fusion or EEG-informed GLMs ([99,103,114], and the fMRI+EEG dataset in [118], with conceptually related multimodal oddball work in [112]). In several cases, the key EEG quantity was not only the averaged P300 but single-trial amplitude fluctuations, which were used to increase sensitivity to trial-by-trial variability in BOLD responses, particularly in pharmacologic or individual-difference contexts [111,112]. Beyond oddball target detection, mismatch paradigms were used to interrogate deviance detection and prediction error processing. A specialized omission MMN task embedded rare omissions into a rapid, highly regular tone stream. At the same time, participants ignored the sounds and watched a silent movie, with event timing synchronized to MRI acquisition to improve EEG–fMRI alignment [102]. An extensive mechanistic study incorporated a conventional duration-deviant MMN as one component of a multi-paradigm battery, alongside rsEEG, rsfMRI, and ASSR, to fit microcircuit DCMs and test a synaptic gain/self-inhibition account across modalities [104]. Other auditory paradigms reduced cognitive demands to separate early from later sensory-perceptual stages, for example, passive listening to repeated tones in a block design; here, joint decomposition of ERPs and fMRI identified N100-linked and P200-linked joint patterns, with group differences emerging primarily in the later P200-linked component [98]. Gamma-band-focused auditory tasks were also included. One study used a cognitively demanding auditory choice–reaction paradigm with sparse-sampling fMRI to quantify the early auditory evoked gamma-band response (aeGBR) and to model trial-wise aeGBR–BOLD coupling as an index of fronto–temporal control network engagement in individuals at high risk for psychosis [106]. Complementing this, the multi-paradigm mechanistic study used a 40 Hz ASSR click–train paradigm to examine induced gamma-band responses and relate them to inferred circuit parameters and symptoms [104].

A smaller set of studies moved beyond audition to target affective context processing, working memory, and semantic integration. One simultaneous EEG–fMRI experiment examined implicit emotional context effects during face perception by presenting a brief, peripheral “crowd” of faces expressing a shared emotion (fearful, happy, neutral, or scrambled) followed by a central target face that participants explicitly rated; this allowed the authors to probe automatic contextual modulation and its network implementation in schizophrenia [96]. Working memory was investigated with a verbal Sternberg task in which participants encoded and retained sets of consonants under low and high load; the core multimodal question was how prestimulus fMRI network states (DMN, dorsal attention, and working-memory networks) predicted retention-period EEG oscillations (theta/alpha/beta), testing state dependency and preparatory dysfunction in psychosis [105]. Semantic mismatch processing was studied with a cross-modal picture–word verification task that elicited the N400 ERP; semantically related versus unrelated mismatches were contrasted, and joint fusion linked N400 difference waveforms to distinct fMRI activation patterns and symptom-relevant network engagement [117].

Finally, two paradigms addressed sensory gating and neuromodulatory manipulation. To study gating in the MRI environment without relying on auditory clicks, one study used somatosensory median-nerve stimulation delivered as single pulses and paired pulses separated by 500 ms or 1000 ms, enabling concurrent measurement of P50 suppression and the fMRI circuitry supporting gating [110]. Another study focused on nicotine’s acute effects in habitual smokers with schizophrenia and healthy smokers using a double-blind crossover design; participants performed a visual oddball choice reaction-time task during simultaneous EEG–fMRI, and an EEG-informed fMRI approach leveraging single-trial P300 amplitudes revealed drug-related increases in ACC/medial frontal activation that were not evident in conventional analyses [111]. Overall, paradigms were selected to combine temporally precise electrophysiological signatures with spatially resolved fMRI measures—either as event-related activations, network dynamics, or resting coupling—so that schizophrenia-related differences could be characterised not only as modality-specific abnormalities but as linked spatiotemporal or network-level dysfunction.

### 3.3. Technical Parameters of Multimodal Neuroimaging

Across studies, “multimodal EEG–MRI” did not refer to a single standardised pipeline but to a family of acquisition and fusion strategies spanning (i) simultaneous EEG–fMRI (by far the most common), (ii) non-simultaneous EEG and fMRI collected in separate sessions but analysed jointly, and (iii) extended multimodal designs that added structural MRI (sMRI) and/or diffusion MRI (DTI) to functional EEG–fMRI measures. Methodologically, studies fell along a continuum from conventional parallel analyses (EEG and fMRI analysed separately) to tightly integrated approaches where one modality directly constrained modelling in the other (e.g., fMRI-informed EEG source/DCM; EEG-informed fMRI GLMs; joint decomposition/factorisation frameworks).

Most work used simultaneous EEG–fMRI on 3T scanners (frequently Siemens Skyra/Trio platforms, but also Philips Achieva in some cohorts). Resting-state simultaneous EEG–fMRI runs commonly lasted ~6–15 min depending on protocol [101,109,113,115], while task runs varied by paradigm (e.g., oddball blocks totalling ~18 min in [99], or shorter runs aligned to event timing and jitter requirements). fMRI acquisition was typically standard EPI BOLD with TRs in the ~2–3 s range (e.g., TR = 2000 ms in [96,100]; TR = 3000 ms in [110,113]; TR ≈ 2992 ms in [102]), with voxel sizes and slice counts tailored to scanner and task. One auditory gamma study adopted a sparse-sampling fMRI design to reduce scanner-noise interference with auditory processing and to create gradient-artefact-free EEG segments for high-frequency analyses [106]. In contrast, at least two task paradigms collected EEG/ERP and fMRI in separate sessions (same day or separated by ~2 weeks on average) while keeping the stimulus design constant, then fused features across modalities offline [114,116,117]. This “sequential multimodal” approach avoided the technical burden of EEG in the scanner but retained cross-modal linking at the level of subject-specific response patterns.

EEG acquisition inside MRI was dominated by MR-compatible caps ranging from ~30 to 32 channels (e.g., [98,101,102,110,115]) through ~62 to 66 channels (e.g., [96,100,106,114]) up to high-density 92-channel montages in some Bern datasets ([105,109], and reduced-to-31-channel analyses for cross-site harmonisation in [107]). To support robust removal of MRI artefacts, EEG was usually sampled at very high rates—most commonly 5 kHz—with scanner clock synchronisation and auxiliary ECG (and often EOG) channels to model cardioballistic effects. A notable exception was an eyes-open resting EEG dataset acquired outside the scanner context in a three-modality fusion study, recorded at 1 kHz for 5 min (eyes open) and fused later with rsfMRI and sMRI features [97]. Some multimodal oddball fusion studies represented EEG as event-related potentials (ERPs) organised as a third-order tensor (subjects × time × electrodes), enabling multilinear decomposition/factorisation methods that preserve EEG’s natural structure [99,103].

Because EEG recorded during fMRI is dominated by scanner-related artefacts, preprocessing pipelines consistently included gradient artefact correction (often template subtraction/average artefact subtraction) and cardioballistic (BCG) correction (ECG-informed subtraction or pulse-template methods). Many pipelines then applied ICA to remove residual ocular, muscle, and scanner-related components (e.g., [96,100,101,102,105,106,109,110,115]). Several studies added more specialised denoising steps; canonical correlation analysis (CCA) was used in some pipelines to suppress broadband/EMG-like contamination in simultaneous EEG–fMRI [98,101,115], and automated artefact screening tools (e.g., FASTER) were used to flag problematic segments or TR-epochs [101,115]. After cleaning, EEG features were extracted in forms matched to the study’s fusion approach: classic ERP peak amplitudes/latencies (P1/N170; N100/P200; N2/P3/P300; N400; P50), single-trial ERP amplitudes (notably P300), band-limited power or synchronisation metrics (gamma power, residual alpha, global field synchronisation), and in some resting studies directed or propagating dynamics (e.g., directed transfer measures and traveling waves).

On the MRI side, the multimodal signal was usually fMRI BOLD, but several studies incorporated additional structural information. Structural MRI (grey-matter maps) was included either as a third modality in fusion/classification contexts [97,98] or in fMRI+sMRI multimodal CCA comparisons [118]. White-matter microstructure (DTI) was explicitly paired with simultaneous EEG–fMRI in an omission-MMN study to relate functional measures to tract-level FA/MD and TBSS results, providing a three-way structure–function linkage across electrophysiology, hemodynamics, and white matter [102]. In resting-state and network studies, fMRI processing frequently emphasised nuisance control (motion regression, WM/CSF regression, aCompCor), temporal filtering in the low-frequency band (often 0.01–0.1 Hz), and either voxelwise analyses or ROI/network-based approaches (DMN seeds, triple-network ROIs, cortical gradient embeddings).

Importantly, what most distinguished studies was not only acquisition but the multimodal integration technique used to couple EEG and MRI-derived measures. A first class used EEG-informed fMRI models, where trial-to-trial or epoch-to-epoch EEG metrics entered fMRI GLMs as parametric modulators to explain BOLD fluctuations beyond event timing alone; this approach was central in studies linking gamma or P300 variability to BOLD networks and pharmacologic effects [101,106,107,111,115]. A second class used fMRI-informed EEG modelling, exemplified by fMRI-constrained EEG source analysis and fMRI-informed EEG dynamic causal modelling (DCM), where fMRI-defined regions guided EEG network modelling and practical connectivity estimation [96]. A third class emphasised data-driven fusion/decomposition, including joint independent component analysis (jICA) combining ERP waveforms with fMRI contrast maps to extract coupled spatiotemporal components that differentiate groups [98,114,116,117] and canonical correlation-based methods such as mCCA and multi-set CCA to identify cross-modal components with correlated subject-wise expression [97,118]. Finally, several papers leveraged matrix–tensor fusion frameworks (CMTF/ACMTF) that explicitly preserve EEG’s multilinear structure while coupling modalities through a shared subject mode, sometimes extending fusion to include sMRI; these methods were used primarily in oddball datasets and evaluated for interpretability and discrimination [99,103].

### 3.4. Early Sensory and Perceptual Processing

Early sensory and perceptual abnormalities in schizophrenia were not uniformly expressed as simple amplitude reductions in EEG or as robust whole-brain fMRI contrasts; instead, the most consistent findings emerged when studies interrogated how early sensory signals are integrated into network dynamics—via fMRI-informed EEG modelling, joint decomposition, or cross-modality structure–function linkage. Across paradigms, a recurring pattern was that very early evoked responses (e.g., P1/N100/MMN-range measures) were sometimes preserved or only subtly altered, whereas later perceptual–integrative stages (e.g., N170 context modulation; P200; higher-order feedback to primary sensory cortex) more reliably differentiated patients and controls.

#### 3.4.1. Visual Perceptual Processing and Affective Context Modulation

Only one study in this set directly probed early visual processing in a perceptual task while linking EEG and fMRI. In the face–crowd paradigm, both groups viewed a peripheral “crowd” of faces (fearful, happy, neutral, or scrambled), followed by a central target face that participants rated; the crowd was task-irrelevant by design, enabling measurement of implicit contextual modulation [96]. At the EEG level, patients exhibited reduced P1 amplitude time-locked to the target face, consistent with diminished early visual processing. The N170 did not primarily express a schizophrenia-vs-control separation; instead, it showed a main effect of crowd condition, with smaller N170 amplitudes when targets followed fearful or neutral crowds compared with scrambled crowds, implying early face-selective processing was sensitive to context but not strongly group-specific in scalp ERPs [96]. Whole-brain fMRI likewise produced primarily condition effects (e.g., fusiform/visual cortex and insula involvement) with limited corrected group-level contrasts, suggesting that standard activation maps were not sufficient to isolate schizophrenia-specific alterations under stringent correction.

The schizophrenia-relevant result emerged at the network-mechanistic level; using fMRI-defined regions to constrain EEG source modelling and fitting EEG dynamic causal models to ERPs, the authors identified a fear-specific group-by-condition difference in top-down feedback from left occipital fusiform gyrus to primary visual cortex (OFG → V1) [96]. For targets preceded by fearful crowds, patients showed enhanced/positive backward connectivity, whereas controls showed negative connectivity, indicating an abnormal context-dependent re-entrant influence on early visual cortex. Significantly, the strength of this fear-context top-down coupling correlated with alexithymia (TAS-20) in patients, linking an early perceptual feedback mechanism. Taken together, these findings support an event-affective phenotype [86]. In this dataset, then, the most “early” schizophrenia effect was not simply reduced occipital ERP amplitude or focal fMRI hypoactivation, but a context-specific disturbance of sensory-cortical feedback control that became apparent only through multimodal effective-connectivity modelling.

#### 3.4.2. Auditory Evoked Processing: N100 Preservation and P200-Linked Divergence

Auditory paradigms provided a richer set of early sensory findings. In passive tone listening with simultaneous EEG–fMRI and joint ICA, two interpretable joint components corresponded to canonical auditory ERP peaks: an N100-linked component and a P200-linked component [98]. The N100-linked component showed the expected coupling between an N100 ERP time window and bilateral temporal auditory cortex activation. Still, it did not significantly differ between schizophrenia-spectrum patients and controls, suggesting that the earliest stage of auditory cortical response to simple tones may be relatively preserved in at least some samples when measured through a cross-modal covariation framework [98]. In contrast, the P200-linked joint component separated groups; controls expressed a larger P200-linked pattern than patients. The fMRI side of this component captured a broader network relationship in which stronger P200 expression covaried with increased activity in bilateral temporal auditory cortex, together with frontal–parietal regions (and cerebellar clusters). In contrast, reduced P200 expression covaried with relatively greater default-mode midline and visual/occipital involvement. Clinically, smaller P200-linked expression was strongly related to greater avolition/apathy (SANS global avolition/apathy) within the patient group [98]. This study, therefore, positioned the key schizophrenia-related deviation not at the earliest sensory response (N100), but at a later perceptual–attentional stage (P200), reflecting how auditory processing recruits broader integrative networks.

#### 3.4.3. Mismatch and Prediction Error in Early Auditory Time Windows

Mismatch negativity (MMN)-range paradigms yielded mixed evidence for a straightforward early sensory deficit when measured at the scalp, but more substantial evidence for altered temporal-lobe engagement and structure–function relationships.

In the omission MMN study with simultaneous EEG–fMRI, the omission response in EEG showed an MMN-range component in both groups. Yet, between-group differences in MMN amplitude/latency were not significant after adjusting for covariates (age and education) [102]. fMRI, however, identified reduced omission-related BOLD response in temporal cortex (notably left MTG in whole-brain comparison, and bilateral MTG effects in ROI analyses), indicating that early deviance processing had an observable hemodynamic signature even when scalp MMN differences were weak in this sample [102]. The multimodal emphasis broadened further with DTI; voxelwise TBSS showed widespread FA reductions, including cingulate-related clusters, and the paper reported associations between cingulate tract metrics and functional measures (e.g., relationships between ACC FA and STG BOLD signal change, alongside symptom associations) [102]. The overarching message was that early mismatch processing abnormalities may manifest more reliably as temporal-cortical BOLD alterations and connectivity/white-matter links than as large scalp MMN group differences in some cohorts—especially small samples with covariate differences.

A complementary perspective came from the extensive mechanistic modelling study that included MMN alongside rsEEG, ASSR, and rsfMRI. There, conventional analyses replicated reduced MMN mismatch responses in patients (and relatives), and DCM-based inference attributed MMN deficits primarily to altered microcircuit “gain” (increased self-inhibition) in frontal nodes (e.g., IFG) in the mismatch contrast, embedding an MMN-range effect within a broader cross-paradigm account of altered synaptic gain control [104]. Although this work was not a single-task simultaneous fusion in the same way as [102], it underscored that “early sensory” abnormalities can be more stable when interpreted through circuit parameters shared across modalities rather than through a single ERP amplitude summary.

#### 3.4.4. Sensory Gating as a Perceptual Filtering Mechanism

The somatosensory gating study extended early sensory processing from stimulus registration to filtering/suppression of repeated input, using median-nerve stimulation to avoid auditory-click confounds in the MRI environment [110]. A practical challenge was that a sizeable subset of participants lacked a clearly identifiable P50 waveform, reducing analysable EEG samples (13 patients; 15 controls) and thereby limiting sensitivity. Within analysable subjects, controls showed evidence of gating (suppression) primarily in the 500 ms paired condition, while patients did not show significant suppression at either 500 or 1000 ms; however, the overall group effect on P50 ratio was not statistically robust in the main ANCOVA framework [110]. fMRI nonetheless demonstrated reliable engagement of somatosensory networks and highlighted patient-specific effects such as ipsilateral deactivation during the 500 ms paired condition and condition-dependent group differences in somatosensory/parietal regions.

Crucially, the multimodal contribution was the linkage between the P50 gating index and BOLD activation in hypothesised gating circuitry; across participants, better gating (lower S2/S1 ratio) correlated with stronger single-stimulus activation in regions including hippocampus, thalamus, STG, and left IFG pars opercularis—supporting the interpretation that these regions contribute to the gating mechanism even when group differences in the scalp ratio are equivocal in small samples [110]. Thus, the key early-perceptual insight was less a reliable case–control separation in P50 itself and more a cross-modal mapping of a classic EEG gating marker onto a distributed fMRI gating circuit.

#### 3.4.5. Synthesis: What “Early” Means in Multimodal EEG–MRI

Taken together, early sensory and perceptual results across modalities suggest that schizophrenia-related differences are often not maximised at the earliest cortical response (e.g., N100 or MMN amplitude) in every dataset, but appear more consistently as (i) disturbances in top-down or integrative control over early sensory cortex (fear-specific OFG → V1 feedback in [96]), (ii) later perceptual–attentional components that still sit close to perception (P200-linked joint patterns in [98]), and (iii) structure–function or EEG–BOLD mappings that reveal abnormal circuitry engagement even when scalp markers are weak (temporal cortex BOLD and DTI associations in [102]; gating-circuit correlations in [110]). In this literature, multimodal methods therefore shift the emphasis from “Is the early ERP smaller?” to “How is early sensory processing embedded within—and regulated by—distributed networks and anatomical pathways in schizophrenia?”

### 3.5. Cognitive Control, Salience, and Target Detection Networks

Across the multimodal EEG–MRI literature reviewed here, the most reproducible schizophrenia-related effects emerged during paradigms that demand attentional selection, salience-driven reorienting, and target detection—most commonly oddball-style tasks. In this domain, unimodal findings (reduced P300, broadly reduced task fMRI activation) were often already apparent. Still, multimodal integration sharpened the interpretation by identifying which networks are specifically “uncoupled” from electrophysiological markers of target processing, and by revealing drug- or symptom-relevant effects that were weak or invisible in conventional analyses.

#### 3.5.1. Oddball Target Detection: Robust Late ERP Deficits with Network-Specific BOLD Coupling Loss

The clearest multimodal convergence concerned the P3/P300 time range, a canonical index of context updating and target evaluation. In a visual oddball study that separated EEG/ERP and fMRI sessions but fused them via joint ICA, schizophrenia patients showed relatively preserved early sensory components (P100/N100) yet a marked reduction in P300 amplitude to targets, alongside reduced target-related fMRI activation distributed across attention systems [116]. Fusion was decisive; the single joint component differentiating groups coupled a P300-like ERP waveform to a spatial map centred on the ventral attention/salience network, including anterior cingulate cortex (ACC), anterior insula, and temporo-parietal junction (TPJ). Patients expressed this coupled P300–ventral-network component substantially less than controls, supporting the conclusion that oddball impairment is not primarily an early sensory failure, but a disruption of salience/reorienting circuitry linked to the P300 process [116].

A conceptually similar dissociation between early and late processes also appeared in an auditory oddball dataset described in the abstracts; patients showed reductions in P300 amplitude and reduced evoked delta/theta/alpha power, and critically, healthy controls showed expected correlations between P300 amplitude and BOLD activation in target-detection-relevant regions (e.g., superior parietal/praecuneus), whereas these electrophysiology–hemodynamic correlations were absent in patients [112]. Although this study reported additional ICA analyses relating networks to reaction time in both groups, the key schizophrenia signal again was a breakdown of normal P300–BOLD coupling rather than a simple lack of task activation per se [112].

Together, these studies point to a specific multimodal signature: schizophrenia target-detection deficits align with reduced late electrophysiological responses and reduced or absent recruitment/coupling of salience and attentional networks that usually scale with those late EEG markers.

#### 3.5.2. N2-Range Coupled Abnormalities and Fronto–Temporal Circuitry

Not all multimodal oddball signatures were centred on P300. In an auditory oddball study using joint ICA to fuse an fMRI target-contrast map with an ERP waveform (Cz) acquired in separate runs, only one joint component robustly differentiated chronic schizophrenia outpatients from controls. This component expressed an ERP pattern concentrated in the N2 time range (~180–200 ms) and an fMRI spatial pattern centred on bilateral fronto–temporal circuitry (including superior/middle temporal and frontal regions), with reduced expression in patients [114]. This suggests that, in some datasets, the strongest coupled abnormality may occur at the earlier evaluation stage indexed by N2—often linked to deviance detection, target categorisation, and cognitive control—while still implicating an extended fronto–temporal network critical for auditory target processing [104].

mCCA-based fusion of an auditory oddball EEG–fMRI dataset reinforced the presence of N2-dominant cross-modal components. The strongest discriminating mCCA pairs linked temporal-lobe and cingulate/motor fMRI patterns to ERP waveforms dominated by a pronounced N2 peak, with significant patient–control differences in the subject profiles for several component pairs [118]. Notably, one mCCA pair demonstrated a modality-asymmetric group effect (ERP profile differing by group without a parallel fMRI profile difference), illustrating how flexible coupling approaches can detect selective electrophysiological disruption even when the corresponding fMRI pattern is less discriminative under the exact decomposition [118].

Taken together, these N2-focused results complement the P300–salience findings by indicating that schizophrenia-related target-detection abnormalities can appear as a coupled deficit either at later context-updating stages (P300) or at mid-latency evaluation stages (N2), depending on task design, features used for fusion, and sample characteristics.

#### 3.5.3. EEG-Informed fMRI and Pharmacologic Modulation of Control Circuitry

EEG-informed fMRI approaches—where trial-by-trial EEG features modulate fMRI regressors—were particularly valuable for detecting subtle influences on control systems. In a nicotine challenge study (smokers with schizophrenia vs. healthy smokers) using simultaneous EEG–fMRI during a visual oddball choice reaction-time task, conventional behavioural and averaged EEG measures showed limited sensitivity to drug effects. There was no robust diagnosis-by-drug interaction at the behavioural level. However, when single-trial P300 amplitudes informed the fMRI model, nicotine produced significant increases in task-related activation concentrated in ACC/medial prefrontal cortex in both groups; this effect was not apparent in conventional fMRI modelling and did not manifest as a clear patient–control difference in nicotine response under cluster-corrected whole-brain analyses [111]. Methodologically, this demonstrates that multimodal integration can reveal state- or drug-dependent engagement of control circuitry even when classic unimodal markers (mean P300 amplitude, RT) do not change appreciably.

#### 3.5.4. Data-Driven Fusion Frameworks: Preserving EEG Structure Improves Interpretability of Cognitive-Control Components

Several studies emphasised that the interpretability of multimodal cognitive-control signatures depends on how the EEG is represented and fused. In auditory oddball datasets where EEG was modelled as a tensor (subjects × time × electrodes), coupled matrix–tensor factorisation (ACMTF) extracted fused components whose EEG time courses resembled canonical oddball features (P2–N2 transitions, P3 peaks) and whose fMRI maps implicated sensorimotor/parietal systems showing group differences; these components also supported relatively high patient–control discrimination and remained stable in leave-one-out robustness tests [99]. Closely related work explicitly compared electrode configurations and found that a targeted mid-size electrode set could yield clearer fused components than using too few electrodes (insufficient information) or too many (noisy, reduced interpretability), with schizophrenia–control differences expressed in fused components linking N2–P3/P3-like EEG dynamics to fMRI patterns including DMN- and parietal/visual-related regions [103]. Although these studies were often framed partly around method validation, the substantive neurobiological implication is that schizophrenia-related differences in oddball processing can be summarised as linked spatiotemporal components rather than separate “ERP deficit” and “BOLD deficit” findings.

#### 3.5.5. Summary: Multimodal Evidence for Disrupted Salience-Driven Control During Target Detection

Overall, multimodal EEG–MRI results in target detection converge on two complementary mechanisms. First, schizophrenia is characterised by reduced late electrophysiological signatures of target evaluation (especially P300) coupled to decreased expression of a ventral attention/salience network component anchored in ACC and anterior insula, consistent with impaired stimulus-driven reorienting and salience assignment ([116], with convergent coupling-loss evidence in [112]). Second, some datasets highlight earlier coupled abnormalities in the N2 time range tied to fronto–temporal circuitry, suggesting impairment in intermediate target/deviance evaluation stages [114,118]. Across approaches, multimodal integration repeatedly reframed “reduced activation” into a more specific statement: schizophrenia involves a breakdown in the normal linkage between electrophysiological target-processing markers and the recruitment of the large-scale networks that implement salience detection and cognitive control.

### 3.6. Working Memory and State-Dependent EEG–fMRI Relationships

#### 3.6.1. Prestimulus Network Preparation Predicts Retention Oscillations in Controls, but Is Reorganised in Psychosis

The central WM study used a verbal Sternberg task during simultaneous EEG–fMRI to examine how prestimulus fMRI network activity relates to EEG oscillations during the subsequent retention interval [105]. Participants encoded either two or five consonants, maintained them over a 3.5 s retention period, and responded to a probe, allowing separation of preparatory/prestimulus, encoding, and retention phases. On the fMRI side, the analysis focused on time courses of four temporally coherent networks (TCNs): the default mode network (DMN), dorsal attention network (dAN), and left and right working-memory networks (WMNs) extracted using template-based spatial–temporal regression. On the EEG side, retention-period spectral amplitude in theta (3–7 Hz), alpha (8–12 Hz), and beta (13–20 Hz) bands was quantified after MRI artefact correction.

In healthy controls, prestimulus network dynamics showed the canonical preparatory organisation expected for efficient cognition: DMN activity was suppressed, and dAN activity increased prior to demanding trials, consistent with a shift from internally directed processing toward externally oriented attention control [105]. Crucially, this prestimulus network configuration predicted subsequent retention-period oscillations. The planned multimodal test emphasised the DMN–theta relationship at high load; controls showed an inverse coupling such that greater prestimulus DMN activity predicted lower frontal-midline theta during retention. This pattern aligns with the study’s framing that successful preparation involves DMN suppression that facilitates WM-relevant theta dynamics later in the trial [105].

Patients with psychotic-spectrum diagnoses showed a different profile at each level of this state-dependent chain. Behaviourally, they exhibited the expected WM impairment pattern: overall slower responses and a load-dependent accuracy reduction, with the most significant deficit at the higher memory load [105]. At the fMRI network level, patients displayed a less robust “push–pull” relationship between DMN and dAN during preparation, characterised by reduced DMN suppression and reduced dAN activation in the prestimulus period, particularly under higher load demands. During retention, patients also showed altered recruitment of WM networks, with comparatively greater right-hemisphere WMN involvement than controls, interpreted as less efficient or compensatory allocation [105].

#### 3.6.2. Load-Dependent Oscillatory Scaling Is Consistent in Controls but Heterogeneous in Patients

Beyond state coupling, the study also examined whether retention oscillations scaled reliably with increasing load. Controls showed topographically consistent increases from load 2 to load 5 in theta, alpha, and beta during retention, including a frontal-midline theta component consistent with WM control signals [105]. In contrast, patients did not show topographically consistent load modulation in theta/alpha/beta, implying weaker or more heterogeneous oscillatory scaling with cognitive demand. This difference matters for multimodal inference; if oscillatory responses do not scale consistently across individuals in the patient group, then any “average” EEG effect is less informative, and cross-modal coupling measures become especially valuable to capture reorganised relationships between brain state and oscillatory dynamics [105].

#### 3.6.3. Multimodal Covariance Mapping Reveals Altered DMN–Theta Coupling Topology in Psychosis

The defining multimodal result was that the prestimulus network–retention oscillation relationship itself was altered in schizophrenia-spectrum participants. For the key DMN-to-theta relationship at high load, controls exhibited a reliable inverse coupling with a frontal-midline theta topography. In contrast, patients did not show the same planned frontal-midline pattern. Moreover, when both groups exhibited consistent covariance patterns, the topography of the DMN–theta covariance differed significantly between groups, described as more spatially extended and left-lateralised in patients. In other words, psychosis was associated not merely with “weaker coupling,” but with a qualitatively reorganised coupling map linking DMN state to theta dynamics [105]. This is a concrete example of how multimodal analysis moves beyond detecting abnormal activation or abnormal oscillation in isolation to detecting abnormal rules of interaction between large-scale networks and electrophysiological control processes.

The study also reported suggestive state-dependency differences for other network–band relationships, such as patterns where patients’ lower-load dAN–theta coupling resembled controls’ higher-load pattern—interpreted as needing greater attentional preparation even at modest demand—though these effects were less emphasised than the DMN–theta finding [105]. Importantly, medication dose and symptom severity did not straightforwardly account for the covariance-map differences, supporting the interpretation that altered state dependency reflects a core physiological feature rather than a simple medication artefact in this dataset [105].

#### 3.6.4. Implications: WM Dysfunction as Disrupted Preparation-to-Maintenance Coupling

Taken together, multimodal evidence from the WM paradigm supports a specific mechanistic framing: schizophrenia-related WM deficits may arise in part from abnormal preparatory network states (insufficient DMN suppression and attentional engagement) that fail to set up or appropriately modulate the oscillatory dynamics needed during retention. The critical abnormality is therefore not only reduced behavioural accuracy at higher load or altered retention activation, but an altered cross-modal dependency linking what the brain is doing before encoding to how it sustains information during maintenance. This state-dependent coupling perspective is challenging to capture with unimodal analyses alone and represents a distinctive contribution of simultaneous EEG–fMRI to understanding WM dysfunction in schizophrenia-spectrum illness [105].

### 3.7. Resting-State Coupling: Frequency Specificity, Directionality, and Timing

Resting-state multimodal EEG–fMRI studies converged on the idea that schizophrenia is characterised not simply by altered “resting activity,” but by altered coupling rules between electrophysiological dynamics and BOLD signals—expressed as changes in frequency specificity (which bands modulate which circuits), directionality (bottom-up vs. top-down propagation), and timing (canonical vs. noncanonical EEG–BOLD lag structure). A consistent motif was that group differences often appeared in EEG–BOLD coupling maps even when mean EEG measures did not differ, indicating that the abnormality lies in how neural fluctuations are translated into hemodynamic/network dynamics rather than in band power alone.

#### 3.7.1. Frequency-Specific EEG–BOLD Coupling in Auditory and Frontal Systems

One line of work treated resting EEG features as time-varying physiological regressors and tested where BOLD fluctuates with those features. A study motivated by the idea that scanner acoustics make “rest” partially stimulus-like focused on two EEG measures computed per TR: low-gamma power (30–50 Hz) and the aperiodic 1/f exponent (estimated with FOOOF) [101]. Critically, schizophrenia-spectrum participants and controls did not differ in mean gamma power or mean aperiodic exponent, and gamma and the aperiodic slope were similarly related across groups. Despite this, coupling differed: schizophrenia-spectrum participants showed reduced gamma–BOLD coupling in bilateral superior temporal gyrus (auditory cortex), with a significant cluster in right STG (cluster-level FDR *p* ≈ 0.038) and a near-threshold left STG effect (*p* ≈ 0.052). The aperiodic exponent showed a separate coupling abnormality in the left superior frontal gyrus/supplementary motor area, with opposite coupling sign across groups (negative in controls, positive in schizophrenia-spectrum; cluster-level FDR *p* ≈ 0.01) [101]. Significantly, within the patient group, weaker right STG gamma–BOLD coupling correlated with worse self-reported sensory gating and higher overall symptom severity, tying an intrinsic coupling measure to clinically meaningful sensory and global symptom burden [101]. In this framework, resting abnormalities were expressed primarily as where and how fast activity fluctuations couple to BOLD, rather than as elevated or diminished gamma power per se.

#### 3.7.2. Global Synchronisation Coupling and Sign Reversals in DMN Hubs

A complementary resting approach emphasised large-scale EEG integration via Global Field Synchronisation (GFS) and asked where BOLD fluctuations covary with GFS fluctuations in standard bands. In a two-site simultaneous EEG–fMRI study combining cohorts from San Francisco VA and Bern, group differences emerged in delta, alpha1, and beta bands, with substantial delta effects in default-mode regions [107]. The key qualitative pattern was a reversal of coupling direction: in multiple DMN-related clusters (praecuneus/posterior cingulate/inferior parietal territory), psychotic patients showed negative coupling where controls showed positive coupling (delta), and in a praecuneus/cuneus cluster (beta), patients again showed coupling of opposite sign to controls. Alpha1 band differences were localised to left occipital/temporal/parietal (extrastriate visual) regions, again with opposing group tendencies [107]. The authors’ control analyses suggested these effects were not reducible to global EEG power modulators, supporting a distinction between coupling to phase synchronisation versus coupling to amplitude.

Together with the gamma–BOLD work, these findings reinforce that resting-state abnormalities in psychosis can manifest as band- and metric-specific coupling disturbances (synchronisation vs. power; oscillatory vs. aperiodic), often involving DMN hubs and sensory cortex.

#### 3.7.3. Rhythm-Dependent Modulation of Thalamo–Cortical and Triple-Network Interactions

Another frequency-specific line treated EEG rhythms not merely as correlates of BOLD but as modulators of directed functional interactions among resting-state networks. In a significant simultaneous resting EEG–fMRI study, time-varying “dynamic rhythm information” was derived using adaptive directed transfer methods across canonical bands (delta, theta, alpha, beta, gamma). These rhythm time series entered PPI-style models to estimate how connectivity among thalamic subregions and triple-network ROIs (DMN/SN/CEN) depends on rhythm fluctuations [100]. The reported schizophrenia-related effect was not simply reduced connectivity but altered rhythm specificity: several directed relationships (e.g., THA → DMN, DMN → SN, SN → CEN) were rhythm-specific in controls but described as not rhythm-specific in schizophrenia, implying a loss of frequency-selective modulation of inter-network signalling. At the network-synergy level, controls showed relatively stronger “thalamus-centred” coupling states, whereas schizophrenia showed a reversal toward greater “CEN-centred” coupling. Additional analyses identified rhythm-conditional coupling differences (e.g., altered DMN–SN coupling under beta/gamma; altered thalamus-related coupling under alpha/theta) and selective associations with onset age and illness duration in particular rhythm conditions [100]. In this formulation, resting dysfunction was expressed as a disruption of how rhythms gate network interactions, consistent with a “loss of selective coordination” account rather than a uniform weakening.

#### 3.7.4. Directionality: Traveling Waves Linked to fMRI Hierarchy and Fronto–Occipital Organisation

Moving beyond frequency specificity, one study operationalised directionality using EEG traveling waves and linked this to fMRI-derived hierarchical organisation. Resting fMRI functional connectivity gradients were used to compute “gradient eccentricity” (a measure of hierarchical segregation in low-dimensional gradient space), while EEG traveling waves along posterior-to-anterior midline electrodes quantified forward (occipital → frontal; putatively bottom-up) and backward (frontal → occipital; putatively top-down) propagation in delta and theta bands [108]. Schizophrenia showed two coupled signatures: (i) altered hierarchy measures, including reduced fronto–occipital hierarchical separation, and (ii) altered directionality dynamics, notably increased theta forward-wave power. Crucially, the cross-modal coupling between hierarchy and directionality was reorganised. Compared with controls, schizophrenia showed stronger coupling between gradient eccentricity and forward-wave power in frontal/association regions and weaker coupling in occipital regions (for both delta-FW and theta-FW), with additional differences involving backward-wave coupling patterns that repeatedly implicated the insula [108]. Symptom dimensions and illness duration moderated parts of these hierarchy–wave relationships, suggesting that directional coupling measures may track clinically relevant heterogeneity rather than serving only as group classifiers [108]. This work reframed “fronto–occipital dysconnectivity” as a disturbance of hierarchical segregation plus altered bottom-up signalling, rather than a static reduction in FC.

#### 3.7.5. Timing: Canonical and Noncanonical EEG–BOLD Lags and Schizophrenia-Related Delay

A distinct resting contribution came from explicitly modelling the temporal lag structure between EEG fluctuations and BOLD, instead of assuming a fixed hemodynamic response delay. Using residual alpha power at Pz (alpha above the fitted aperiodic component), one study computed alpha–BOLD correlations across multiple lags (−10 to +10 s in 2 s steps) and summarised each region’s lag bias via an “Accumulated Correlation Asymmetry” (ACA) metric [115]. Two key results followed. First, alpha–BOLD coupling exhibited noncanonical timing, especially within the DMN: posterior DMN regions tended to peak at very short lags (near 0–2 s), whereas anterior DMN regions peaked closer to canonical ~4–6 s delays. Second, schizophrenia was associated with more delayed/right-shifted coupling (higher ACA) in a subset of regions, including the superior frontal cortex, parahippocampal gyrus, fusiform gyrus, angular gyrus, praecuneus, pregenual ACC, and brainstem. These timing effects were not explained by residual alpha amplitude (no group difference), and were not strongly attributable to medication, BMI, or heart rate in the reported analyses [115]. This study, therefore, positioned resting abnormalities partly as a temporal misalignment of electrophysiological–hemodynamic coupling, potentially contributing to inconsistencies in studies that fix the lag a priori.

#### 3.7.6. RSN–EEG Frequency “Downshift” and Reduced Coupling Consistency

Another resting-state line focused on linking EEG spectral dynamics to identified resting-state networks (RSNs) via covariance mapping. In a small schizophrenia-spectrum inpatient cohort and matched controls, DMN and a left working-memory/language network (LWMN) were extracted via ICA, and EEG spectral amplitude dynamics were coupled to RSN time courses to produce topographic covariance maps across multiple frequency bands [109]. Patients produced fewer frequency bands with consistent covariance topographies (i.e., less reliable RSN–EEG coupling patterns across subjects). Only one within-band topographic group difference reached significance (LWMN–theta1 covariance topography). Still, the study’s central observation was a cross-frequency shift: patient covariance maps at lower frequencies resembled control covariance maps at higher frequencies (e.g., DMN patient theta2/alpha2 resembling control beta1; LWMN patient theta resembling control alpha/beta). This “downshifted” coupling was not explained by antipsychotic dose in topographic ANCOVA tests within patients [109]. Although modest in sample size, these results align with broader themes that schizophrenia alters not only within-band power but also the frequency channel through which RSNs couple to electrophysiology.

#### 3.7.7. When Multimodal Rest Shows Weak Within-Network Differences

Not all resting multimodal studies found strong within-network abnormalities. In a small simultaneous resting EEG–fMRI study focused on the DMN, seed-based rsfMRI and EEG source-space lagged coherence analyses did not show robust within-DMN group differences but did identify increased rsfMRI connectivity between an mPFC DMN node and a right posterior inferior temporal region outside the DMN in schizophrenia [113]. Cross-modality correspondence between fMRI connectivity and EEG lagged coherence was generally weak, underscoring that EEG- and fMRI-derived connectivity measures may capture different physiological aspects of coupling at rest, and that schizophrenia effects may appear more reliably in cross-network or coupling-rule measures than in within-network strength alone [113].

#### 3.7.8. Synthesis: Resting-State Abnormalities as Altered Coupling Rules

Across these resting-state studies, schizophrenia-related differences most consistently appeared as alterations in (i) frequency specificity (which rhythms/synchronisation metrics modulate which networks, and whether that modulation remains selective), (ii) directionality (biases in bottom-up traveling waves and their link to cortical hierarchy), and (iii) timing (noncanonical lag profiles and schizophrenia-related delays). Notably, several results were strongest precisely when unimodal summaries were null or weak—for example, abnormal gamma–BOLD coupling despite no mean gamma differences [101] and delayed alpha–BOLD timing despite no residual alpha differences [115]. This supports a general conclusion of the multimodal resting literature: schizophrenia is often characterised less by a static shift in “resting activity,” and more by a reorganisation of how electrophysiological dynamics map onto hemodynamic networks across frequencies, directions, and timescales.

### 3.8. Extended Multimodality and Clinical Prediction

Beyond simultaneous EEG–fMRI, several studies expanded multimodal designs in two directions: (i) adding anatomical modalities—structural MRI (sMRI) and/or diffusion MRI (DTI)—to link functional abnormalities to brain structure, and (ii) using multimodal feature sets for individual-level discrimination or prediction (most often schizophrenia vs. control classification, and more rarely symptom/phenotype associations). Across these studies, the main pattern was that adding modalities tended to improve separability and interpretability. Still, performance and inference were sensitive to demographic imbalance, sample size, and feature-selection strategy, and clinical prediction was most compelling when multimodal measures reflected mechanistic coupling rather than single-modality summary metrics.

#### 3.8.1. Tri-Modal Structure–Function Integration: EEG+fMRI+sMRI

A prominent classification-oriented example fused resting EEG spectra, rsfMRI ALFF maps, and voxelwise grey-matter maps via three-way multi-set CCA (MCCA) and compared single-modality vs. multimodal SVM classification [97]. The authors reported that the schizophrenia and control groups differed significantly in age and sex, and they addressed this by regressing out age and gender before group tests on component expressions. MCCA identified components whose subject-wise mixing coefficients differed by group across multiple modalities, yielding “modality-common” linked patterns (e.g., EEG spectral features emphasising frontal/prefrontal and occipital/motor channels; grey-matter differences in superior temporal and parahippocampal regions; and ALFF differences in default-mode and frontal/occipital regions). In classification analyses, multimodal feature combinations outperformed single modalities: combining EEG+GM+ALFF was reported to reach ~90% accuracy in cross-validation and to improve sensitivity/specificity relative to single-modality classifiers. A second ensemble selection procedure (combining t-test filtering, MCCA masks, and SVM-RFE) was described as yielding particularly high performance, including a reported 100% prediction rate on repeated 10-subject holdout tests for the whole three-modality combination [97]. Substantively, this study’s contribution was not only accuracy claims but the demonstration that cross-modality covariation can isolate schizophrenia-related patterns that are spatially and spectrally linked, supporting the idea that the disorder is expressed as a coupled alteration across structure, slow BOLD fluctuations, and electrophysiological rhythms.

A separate line of work used structure-revealing coupled matrix–tensor factorisation (ACMTF) to fuse EEG ERPs (as a tensor preserving time × electrode structure) with fMRI task maps, optionally adding sMRI grey-matter features as a third matrix. In an auditory oddball dataset, adding sMRI allowed significant fused components to include structural maps that paralleled functional differences—e.g., components where controls differed from patients in sensorimotor/parietal fMRI activation while also showing greater grey-matter concentration in parietal/cerebellar or frontal/parietal regions depending on component [99]. Methodologically, the study emphasised that sMRI can be challenging to integrate because mean structural variation across subjects can dominate; “subject-centring” of sMRI improved recovery of discriminative structural components and improved clustering performance (reported ~91% clustering accuracy with EEG–fMRI–sMRI ACMTF under subject-centring) [99]. This work thus illustrated a practical point about extended multimodality: structural data can add interpretability and improve discrimination, but only if preprocessing and normalisation prevent sMRI from overwhelming individual-difference signals relevant to diagnosis.

Related EEG–fMRI fusion work underscored that the quality of extended multimodal signatures depends on EEG representation and electrode selection. When preserving EEG’s tensor structure, a mid-size, informative electrode subset (e.g., 11 channels) sometimes produced cleaner, more interpretable fused EEG–fMRI components than using either very few electrodes or very high-density montages that introduced spatial noise in the fused fMRI maps [103]. This has implications for “clinical prediction” workflows: maximising channel count is not necessarily optimal if it reduces the stability/interpretability of fused components.

#### 3.8.2. DTI as a Structural Constraint on EEG–fMRI Functional Measures

One study explicitly connected simultaneous EEG–fMRI measures to white-matter microstructure by adding diffusion MRI (DTI) to an omission MMN paradigm [102]. Although scalp MMN amplitude/latency did not show robust between-group differences after covarying age and education, fMRI revealed reduced omission-related BOLD responses in the temporal cortex (particularly MTG), and TBSS showed widespread FA reductions, including cingulate-related clusters and additional deep white-matter pathways. The paper further reported associations linking functional and structural measures (e.g., relationships between cingulate tract FA and temporal-region BOLD responses, and correlations between cingulum/ACC measures and symptom indices) [102]. The value of adding DTI here was not classification but mechanistic triangulation: abnormalities in temporal mismatch processing were interpreted in the context of altered cingulate/temporal white-matter integrity, consistent with a distributed circuit account rather than a purely local sensory deficit.

#### 3.8.3. Multimodal Discrimination: What Improves and What Limits Generalisation

Across studies that explicitly evaluated discrimination performance, multimodal combinations generally improved separability compared with any single modality, but with notable caveats.

Fusion-based feature extraction (e.g., ACMTF components or MCCA component masks) tended to yield features that were both interpretable and discriminative, often outperforming raw-modality feature sets or EEG-only decompositions [97,99].

Model choices strongly influenced performance; feature-selection steps such as SVM-RFE, stability-based initialisation selection, mask thresholding (e.g., |Z| cutoffs), and the specific cross-validation/holdout design were central determinants of reported accuracy. Some pipelines used repeated cross-validation, while others used repeated small holdouts; the latter can yield optimistic estimates when feature selection is repeated on most of the sample, and testing uses minimal sets [97].

Demographic imbalances and motion were recurring confounds. For example, one classification-oriented multimodal dataset reported significant age and sex differences between SZ and controls, necessitating regression/covariate handling before inference and classification [97]. In other multimodal contexts, patient motion was higher and could influence both fMRI maps and EEG component yield; studies sometimes excluded participants for motion or unusable EEG, which improves data quality but can shift sample composition [98,100,101].

Thus, the literature supports the qualitative conclusion that “multimodal improves discrimination,” but comparisons across studies should be interpreted cautiously because classification estimates are susceptible to design and sample properties.

#### 3.8.4. Clinical Prediction Beyond Diagnosis: Symptoms and Phenotypes

While formal symptom prediction was not the dominant focus of the extended-modality papers, several studies showed that multimodal measures can capture clinically meaningful variation.

In the tri-modal rhythm-modulated connectivity study, specific rhythm-conditional network coupling measures related to age of onset and illness duration, suggesting that resting multimodal dynamics can index clinically relevant heterogeneity rather than only diagnostic status [100].

In the hierarchy/traveling-wave framework, symptom dimensions and illness duration moderated the relationship between fronto–occipital hierarchical separation and theta forward-wave power, indicating that the joint EEG–fMRI measure was sensitive to clinical course and symptom profile [108].

In the omission MMN + DTI study, the authors reported multiple structure–function and structure–symptom associations involving cingulate FA, temporal cortex BOLD, and PANSS components, again illustrating the value of adding a structural modality to contextualise functional coupling measures [102].

These findings suggest that the most promising clinical signals in extended multimodality may not be raw EEG power or fMRI connectivity alone, but cross-modal coupling parameters (e.g., rhythm-dependent connectivity modulation, hierarchy–directionality coupling, structure–function relationships) that can plausibly map onto interpretable circuit mechanisms.

#### 3.8.5. Summary

Extended multimodal studies demonstrate that adding sMRI and/or DTI to EEG–fMRI can (i) provide a mechanistic context for functional abnormalities, (ii) improve interpretability of fused components by linking electrophysiological signatures to both functional activation and anatomical substrates, and (iii) often improve schizophrenia–control discrimination when features are derived from joint models rather than from single modalities. At the same time, reported classification performance depends strongly on feature-selection strategy, sample balance, and validation design. Clinically, the strongest “prediction-relevant” signals tended to arise when multimodal models captured how modalities relate—structure–function coupling, rhythm-dependent modulation, or hierarchy–directionality relationships—rather than when they relied solely on unimodal summary measures.

### 3.9. Symptom Relevance and Medication Sensitivity

A key promise of multimodal EEG–MRI is that it can yield biomarkers that are not only diagnostic but also clinically meaningful—tracking symptoms, functional impairment, or treatment effects. Across the reviewed studies, symptom and medication findings followed a consistent pattern: multimodal coupling parameters (e.g., effective connectivity, joint-component expression, EEG–BOLD coupling strength or timing) were more likely to relate to clinical measures than raw unimodal amplitudes or mean band power. At the same time, medication effects were often examined. They frequently did not provide a simple explanation for group differences, although targeted pharmacologic designs showed that medication manipulation can be detected more sensitively when fMRI is EEG-informed.

#### 3.9.1. Symptom Relevance of Multimodal Coupling and Joint Components

(a) Affective traits and top-down perceptual feedback

The most specific symptom linkage in the set connected an fMRI-informed EEG DCM parameter to an affective phenotype. In the face-context study, schizophrenia patients showed an abnormal fear-specific increase in top-down feedback from left occipital fusiform gyrus to V1 (OFG → V1). Within patients, stronger OFG → V1 backward connectivity in the fearful-context condition correlated robustly with alexithymia (TAS-20), surviving correction for multiple comparisons [96]. This association is notable because (i) behavioural context effects were minimal, and (ii) conventional fMRI contrasts did not yield strong corrected group interactions—suggesting the clinically relevant signal lay specifically in the multimodal effective-connectivity estimate rather than in overt performance or isolated activation.

(b) Motivational symptoms and later auditory processing networks

In passive auditory tone listening with simultaneous EEG–fMRI fusion, the joint ICA component aligned with P200 differentiated groups (controls > patients) and, within patients, lower P200-linked component expression was strongly associated with greater avolition/apathy (SANS global avolition/apathy) after correction. In contrast, the N100-linked component showed neither robust group separation nor comparable symptom linkage [98]. This positions motivational impairment as related to a later-stage auditory–frontoparietal coupling pattern rather than early sensory encoding.

(c) Sensory gating complaints and intrinsic auditory coupling

A resting simultaneous EEG–fMRI study found that mean gamma power and mean aperiodic slope did not differ between schizophrenia-spectrum participants and controls, yet schizophrenia-spectrum participants exhibited reduced gamma–BOLD coupling in the auditory cortex (STG). Within patients, weaker proper STG gamma–BOLD coupling correlated with worse self-reported sensory gating (SGI total) and higher overall symptom severity (PANSS total) [101]. This again highlights a theme: symptoms tracked an abnormal mapping between EEG dynamics and BOLD fluctuations, not the EEG measures averaged across time.

(d) Target detection components and thought content

In a multimodal oddball study combining ERP and fMRI via jICA, the group-differentiating component coupled a P300-like ERP waveform with ventral attention/salience network activation (ACC/insula/TPJ), and within patients, the expression of this joint component was negatively associated with unusual thought content in a post hoc analysis [116]. Although this symptom association was secondary, it is consistent with the idea that salience-network-linked electrophysiological deficits may relate to specific positive symptom phenomenology.

(e) Semantic processing networks and positive symptoms

In the N400 fusion study (ERP–fMRI jICA), schizophrenia patients showed reduced loading weights on the joint N400 component (relative to controls). Still, symptom relationships were more evident in the hemodynamic network engagement than in scalp N400 amplitude. Specifically, unusual thought content was inversely related to extracted fMRI contrast values from N400-linked component maps (for both in-category and out-of-category mismatch maps). In contrast, the joint loading weights themselves and N400 ERP amplitude did not show comparably strong symptom correlations [117]. This dissociation suggests that symptom relevance may reside in which cortical systems support the ERP-defined computation, rather than in the ERP magnitude alone.

(f) Resting network dynamics and illness course

Two resting-state multimodal studies emphasized illness-course and onset relationships. Rhythm-modulated connectivity analyses reported that specific network-coupling measures correlated with age of onset (theta-modulated SN–THA coupling with DMN as output centre) and duration of illness (gamma-modulated DMN–SN coupling with CEN as output centre) [100]. A hierarchy–traveling-wave study found moderation effects whereby positive and negative symptom dimensions and illness duration shaped the relationship between fronto–occipital hierarchical separation and forward/backward traveling-wave power (especially theta forward-wave) in specific frontal, insular, and opercular regions [108]. These findings indicate that multimodal coupling metrics may index clinically meaningful heterogeneity even in resting data, though the specific symptom dimensions implicated differed by method.

(g) Null or weak symptom associations

Not all multimodal measures showed strong symptom coupling. For example, a two-site resting EEG–fMRI GFS coupling study reported robust group differences in coupling direction across DMN clusters but did not find significant relationships between coupling betas and PANSS totals (uncorrected *p* > 0.05) [107]. Similarly, a lagged alpha–BOLD timing study found delayed coupling in schizophrenia in multiple regions but no robust associations with PANSS positive/negative after correction [115]. These null findings are informative: some multimodal markers may reflect trait-like or system-level differences not tightly linked to symptom ratings at the time of scanning.

#### 3.9.2. Medication Sensitivity: When Dose Matters and When It Does Not

##### Dose Is Often Examined, Frequently Not Explanatory

Many cohorts were predominantly medicated, and several studies explicitly tested whether antipsychotic dose explained key multimodal effects. In the RSN–EEG covariance mapping study, within-patient topographic ANCOVA found no significant relationships between chlorpromazine equivalents and covariance-map topography (all *p* > 0.26), suggesting that the observed “frequency downshift” of RSN–EEG coupling was not a simple linear medication-dose artefact in that sample [109]. In the hierarchy–traveling-wave study, chlorpromazine-equivalent dose did not show significant correlations with core eccentricity or wave measures. It did not account for the illness-duration moderation effect in the reported analyses [108]. In the alpha–BOLD timing study, delayed coupling differences were not explained by antipsychotic dose (and other physiological covariates were also examined) [115]. These repeated null results support a cautious interpretation that many coupling abnormalities are not trivially reducible to medication dose, although cross-sectional designs cannot rule out all treatment effects.

##### Specific Medication Associations Can Appear in Selective Coupling Metrics

The rhythm-modulated connectivity study reported one medication-related association: chlorpromazine equivalents correlated with a gamma-modulated coupling measure involving DMN–SN interactions with thalamus as an input centre. However, that coupling did not differ between groups [100]. This type of result suggests that medication may influence particular rhythm-dependent coupling features without necessarily explaining diagnostic differences.

#### 3.9.3. Pharmacologic Challenge Designs: EEG-Informed fMRI as a Sensitive Readout

A nicotine challenge study provides the clearest example of medication sensitivity in a controlled design. In habitual smokers with schizophrenia and healthy smokers, acute nicotine (vs placebo) produced increased ACC/medial prefrontal activation when the fMRI model was informed by single-trial P300 amplitude; conventional fMRI and averaged ERP measures were comparatively insensitive, and no robust diagnosis-by-drug interaction emerged under corrected whole-brain inference [111]. This demonstrates that EEG-informed fMRI can detect subtle pharmacologic modulation of control circuitry even when behavioural measures are unchanged and classic ERP averages do not shift. Practically, it suggests that medication effects may be more detectable when modelled as changes in the coupling between trial-level electrophysiology and BOLD, rather than as changes in mean ERP amplitude or mean activation.

#### 3.9.4. Summary

Across studies, symptom relevance was most consistently observed for multimodal linkage parameters—fear-specific top-down visual feedback linked to alexithymia [96], P200-linked joint auditory network expression linked to avolition/apathy [98], and intrinsic auditory gamma–BOLD coupling linked to sensory gating complaints and overall severity [101]—and for select fusion-defined networks related to positive-symptom phenomena [116,117]. The medication dose was routinely considered and often did not account for core coupling abnormalities. However, selective dose–coupling relationships were occasionally observed [100], and controlled pharmacologic manipulation (nicotine) revealed robust effects primarily detectable through EEG-informed fMRI [111]. Overall, the clinical signal in multimodal EEG–MRI studies of schizophrenia appears strongest when the measure captures how electrophysiology and hemodynamics interact, not merely how large either signal is in isolation.

### 3.10. Risk of Bias Assessment

Risk of bias assessment (ROBINS-I) is presented in Table 2.

## 4. Discussion

Several studies were limited by small sample sizes, medication heterogeneity, or substantial preprocessing complexity, increasing susceptibility to bias and reducing reproducibility. Schizophrenia is a severe mental disorder that requires investigation using various neuroimaging techniques. Multimodal EEG–MRI neuroimaging opens up new perspectives in diagnosing this disorder by combining two methods, each with its own strengths and limitations. The number of studies included in this review demonstrates researchers’ interest in the topic. These studies differ in the neuroimaging techniques, signal analyses, and paradigms used. However, each provides valuable information about the neural mechanisms of schizophrenia. Across the studies, multimodal EEG–fMRI converges on a coherent picture: schizophrenia is not well explained by a single “deficit” in one region or one frequency band, but by state-dependent failures of coordination across hierarchical cortical systems—from early sensory cortex to association networks—expressed as (i) altered top-down vs. bottom-up balance, (ii) abnormal precision/gain control over neural singling, and (iii) disrupted network switching among default-mode, salience, and executive systems. Below, we discuss how the reported findings fit within established mechanistic frameworks and how they may map onto symptoms and behavioural phenotypes.

Interpretation of frequency-specific EEG–fMRI relationships also requires caution. Because the BOLD signal reflects slow hemodynamic responses occurring over seconds, fMRI cannot directly track fast electrophysiological oscillations such as gamma-band activity. Consequently, multimodal findings involving gamma–BOLD coupling should be interpreted as indirect hemodynamic correlates associated with synchronised neural activity rather than direct measurements of gamma oscillations themselves.

Interpretive caution is also warranted regarding anatomical specificity. A major limitation of multimodal EEG–MRI approaches concerns the limited sensitivity of scalp EEG to deep subcortical structures strongly implicated in schizophrenia, particularly the striatum and dopaminergic midbrain systems. Although simultaneous EEG–MRI can capture large-scale thalamo–cortical and cortico-cortical network dynamics, electrophysiological measures obtained from scalp recordings predominantly reflect cortical population activity. Accordingly, multimodal EEG–MRI findings should not be equated with direct measurements of subcortical dopaminergic dysfunction, but rather interpreted as systems-level manifestations of distributed thalamo–cortical and cortico-striatal dysconnectivity.

### 4.1. A Unifying Framework: Dysconnection, Predictive Processing, and Gain Control

A helpful way to integrate the diverse multimodal patterns summarised above is to treat schizophrenia not as a unitary “under-” or “over-activation” syndrome, but as a disorder of context-sensitive coordination—i.e., abnormal regulation of when and how strongly distributed neural populations influence one another. This is the core intuition behind the dysconnection hypothesis: symptoms and cognitive deficits arise primarily from disrupted integration among neuronal systems, driven by abnormalities in synaptic plasticity and the efficacy of connections (rather than gross lesions in any single node) [119,120,121]. Importantly, dysconnection is not limited to anatomical disconnection; it includes aberrant effective connectivity—failures in directed, state-dependent influences among areas—which can manifest even when regional mean activity looks relatively similar between groups [119].

Within this framing, the multimodal results you summarised (across perception, attention, resting-state dynamics, and task preparation) are naturally interpretable as specific instances of dysconnection: the system’s coupling parameters are not globally broken, but are mis-set—often in a condition-specific way (e.g., threat context, task engagement, or particular oscillatory regimes). This aligns with neuroimaging and connectomics accounts emphasising that schizophrenia involves distributed network abnormalities—structural and functional—whose expression depends on brain state and analytic scale [29,30,122]. Large-scale structural evidence supports a substrate on which such dynamic dysconnection can unfold; ENIGMA’s diffusion MRI meta-analysis demonstrates widespread white-matter microstructural differences across thousands of individuals with schizophrenia, providing a plausible anatomical constraint on long-range communication and timing [29]. Yet the same ENIGMA work also underscores that anatomy alone is not destiny—clinical variables like medication dose showed limited explanatory power for the white-matter profile—reinforcing the view that physiological coupling rules (plasticity, gain, neuromodulation) are essential [29].

Predictive processing (predictive coding/active inference) provides a mechanistic computational language for this dysconnection. In hierarchical predictive processing, cortical systems continuously generate top-down predictions about sensory input; mismatches (prediction errors) ascend the hierarchy to update beliefs. Symptoms can be conceptualised as failures of inference—false percepts (hallucinations) or false beliefs (delusions)—emerging when the brain misassigns precision (confidence/weight) to predictions and/or prediction errors [123,124]. In other words, dysconnection becomes computationally specific; it is not merely that areas are weakly connected, but that the messages passed (predictions vs. errors) are weighted incorrectly and therefore drive maladaptive updating. This Bayesian framing has been used to link core psychotic phenomena to abnormal inference and learning signals, including prediction-error-related mechanisms [123,124]. Empirically, human behavioural work shows that hallucination-prone or psychosis-relevant phenotypes can reflect overweighting of prior expectations relative to sensory evidence. This effect fits naturally as a precision imbalance [125].

Crucially, predictive processing is not a purely abstract theory; it maps onto known cortical circuit motifs that are inherently recurrent and layered. Canonical predictive-coding microcircuit models propose that deep-layer pyramidal populations convey predictions via feedback pathways. In contrast, superficial pyramidal populations convey prediction errors via feedforward pathways, with inhibitory interneurons shaping the gain (precision) of these signals [126]. This anatomy makes an immediate bridge to multimodal findings; many of the observed abnormalities are not simple feedforward sensory failures but involve altered feedback control, altered engagement of associative networks during perception, and altered mapping between fast electrophysiology and slow hemodynamics—precisely what one would expect if recurrent message passing and precision control are disrupted.

That bridge becomes even tighter when “gain control” is treated as the physiological implementation of precision weighting. In predictive processing, precision determines the influence of a signal on updating; neurally, precision is widely modelled as a form of postsynaptic gain on the populations encoding prediction errors (and sometimes predictions), controlled by local microcircuit inhibition and neuromodulatory tone [2,124]. Dysconnection accounts explicitly connect schizophrenia to abnormal synaptic plasticity and gain regulation—because if synapses and their context-dependent modulation (e.g., via NMDA-dependent plasticity) are perturbed, then the brain’s ability to tune coupling strengths and precision across contexts will be unstable [119,120]. In practical terms, this predicts a signature that matches current summaries: (i) relatively subtle or absent mean differences in some signals, alongside (ii) marked abnormalities in coupling, directionality, and state dependence—the “rules of interaction” rather than the baseline levels.

Finally, this unifying framework clarifies why schizophrenia can present as a mixture of “too much” and “too little” signalling depending on circuit, task, and symptom dimension. If gain/precision is misallocated, the system may compensate by increasing top-down constraints in some contexts (producing overly strong priors, reduced flexibility, and biased interpretation), while simultaneously failing to amplify genuinely informative sensory prediction errors elsewhere (producing blunted updating, reduced context learning, and impoverished salience assignment) [123,124]. The key implication for interpreting the multimodal EEG–fMRI patterns summarised is therefore not that schizophrenia is globally hypo- or hyper-connected, but that it is mis-calibrated; hierarchical message passing (predictions and errors) is not tuned to context with standard precision control. In the sections that follow, this lens motivates more specific interpretations—fear-specific feedback changes as altered recurrent inference in visual–social perception, late auditory-stage abnormalities as downstream consequences of imprecise updating and network competition, and DMN/salience/thalamic effects as failures of state control that normally gate precision and switching across large-scale networks [119,121,122,124].

### 4.2. Visual Social Perception: Fear-Specific Top-Down Feedback as Altered Recurrent Processing

A central implication of the present results is that schizophrenia-related impairment in social–visual function may not always manifest as a significant behavioural “context effect,” but instead as an alteration in how the visual system implements context through recurrent (feedback) processing—particularly under threat-related (fearful) conditions. In contemporary models of vision, the initial feedforward sweep through the occipital cortex provides a fast, coarse hypothesis about “what is out there,” while subsequent recurrent interactions (feedback and lateral loops) refine that hypothesis by selectively amplifying task-relevant features, suppressing irrelevant signals, and integrating contextual priors with sensory evidence. Conceptually and mechanistically, recurrent processing is therefore the bridge between (i) an inferred scene interpretation and (ii) the low-level sensory representation that must be sharpened to support stable perception and meaningful social judgments.

#### 4.2.1. Recurrent Processing as the Mechanism of Context in Vision

The idea that recurrent loops are essential for integrating context (including attention and meaning) into early sensory representations is long-standing. Classic frameworks distinguish a rapid feedforward pass from a later recurrent phase in which higher visual regions re-enter earlier cortex to support perceptual completion, selection, and conscious report. Lamme’s feedforward–recurrent account explicitly frames recurrent interactions as the mechanism by which vision becomes context-sensitive rather than purely stimulus-driven [127]. This is consistent with empirical demonstrations that perceptual visibility and awareness track dynamic effective connectivity between early visual cortex (including V1) and higher ventral stream regions (including fusiform territories), rather than local activity alone [128,129]. In other words, the “same” feedforward input can yield different precepts depending on whether and how feedback stabilises or suppresses early representations.

In face perception, recurrent processing is essential because faces are complex, socially meaningful stimuli that require rapid extraction of configural structure and fine-grained feature information (eyes, mouth, gaze, micro-expressions) while remaining robust to noise, viewpoint, and low-level ambiguity. Human ventral visual cortex has anatomical and functional properties that support this: higher-level temporal/fusiform regions have feedback projections that can reach back into earlier visual cortex, enabling top-down modulation of sensory coding [128,129]. Moreover, computational and systems-level accounts of visual recognition emphasise that “explaining away” sensory input by higher-level hypotheses is implemented neurologically through recurrent message passing—precisely the sort of top-down influence indexed by feedback connectivity from fusiform/occipito-temporal regions to V1 [130].

#### 4.2.2. Why Fear Is a Special Context for Recurrent Visual Processing?

Fearful facial expressions are not simply another category of emotion; they are threat cues that engage specialised prioritisation mechanisms. Across species, threat processing recruits circuits that can bias perception toward rapid detection and conservative interpretation of ambiguous cues. In humans, converging evidence supports a fast threat-sensitive pathway involving subcortical nodes (notably superior colliculus and pulvinar) interacting with the amygdala, alongside cortical ventral-stream routes [131,132]. Importantly, this architecture is well-suited to modulate cortical visual processing: the pulvinar is increasingly viewed as a hub that regulates information flow across cortical visual systems and supports attention-like gating and coordination, making it an ideal conduit for threat-related state changes to reach visual cortex [131].

At the cortical level, amygdala–visual cortex interactions are commonly implicated in enhanced processing of threat cues and in the allocation of attention toward emotionally salient stimuli. For example, work on visual awareness and emotional faces has shown that amygdala responses—and their coupling with extrastriate regions—depend on perceptual visibility and attentional access, consistent with a role in amplifying or stabilising representations when threat is behaviourally relevant [133,134,135]. More direct neurophysiological evidence indicates that the human amygdala can respond rapidly and preferentially to fearful faces, even under conditions that limit conscious report, supporting the plausibility of early threat signals shaping downstream cortical processing [136,137]. Taken together, fear context is expected to alter the gain and timing of recurrent loops: it can increase the priority of face processing, recruit broader attentional/salience systems, and bias early visual cortex toward features consistent with threat detection.

#### 4.2.3. Schizophrenia and Social–Visual Impairment: From Early Sensory Coding to Face-Specific Circuits

Schizophrenia is reliably associated with deficits in facial emotion recognition, with fear recognition often among the most affected domains. Early behavioural work and large meta-analytic syntheses show robust emotion perception impairments in schizophrenia across tasks and samples [138,139]. More recent meta-analytic work suggests that face perception impairments extend beyond emotion and can involve broader facial judgments, emphasising the need to separate emotion-specific from domain-general contributors [140]. Neurobiologically, these social–visual impairments sit on top of two partly separable vulnerability layers.

The first is early visual processing constraints. There is extensive evidence for early-stage visual abnormalities in schizophrenia, including deficits consistent with magnocellular/dorsal-stream dysfunction and altered contrast gain control. These are not subtle theoretical claims; they are supported by empirical findings and reviews pointing to impairments in early visual cortex function and amplification mechanisms that typically boost weak signals [141,142,143]. Such early constraints matter because they reduce the fidelity of the sensory evidence available to the face system, forcing heavier reliance on priors or top-down filling-in conditions under which abnormal recurrent processing would have amplified consequences.

Second are central-stream and face-network differences. Structural MRI findings indicate that fusiform regions can be reduced in volume early in illness, consistent with altered capacity or efficiency in ventral-stream face processing [144]. Functional neuroimaging meta-analyses of facial emotion processing in schizophrenia report abnormal activation patterns that often include limbic regions (notably amygdala), visual processing areas, and cingulo-frontal control systems, consistent with a distributed network alteration rather than a single “face area lesion” [145,146]. A specific meta-analysis of facial emotion processing has also reported altered activation during fear/anger processing, implicating medial prefrontal/cingulate systems alongside other regions [146].

Within the EEG literature, face-related ERP components provide timing constraints on where recurrent abnormalities might appear. The P1 is commonly interpreted as indexing early sensory–attentional processing in extrastriate cortex, and N170 as an index of structural encoding of faces. A dedicated meta-analysis of face-ERP components in schizophrenia indicates systematic abnormalities in N170/N250 measures, supporting the idea that face-specific processing stages are altered [147,148]. This timing is essential: if early sensory encoding is weakened and face-structural encoding is atypical, recurrent feedback from higher face regions to early visual cortex may be recruited differently—either compensatorily or in a dysregulated manner.

#### 4.2.4. Interpreting Fear-Specific OFG → V1 Feedback: What Does “Stronger Top-Down” Mean?

The key empirical observation to explain is a fear-specific alteration in backward (top-down) coupling from occipito-fusiform cortex to V1. Mechanistically, at least four non-mutually-exclusive interpretations are plausible, and each map onto known properties of recurrent vision.

(a) Compensatory sharpening under noisy sensory evidence

If early visual encoding is degraded (e.g., reduced P1, magnocellular inefficiency, weaker gain control), the system may compensate by increasing top-down sharpening—essentially “pushing” a higher-level face hypothesis back to V1 to stabilise uncertain features. This is consistent with broader evidence that schizophrenia involves early-stage visual constraints [141,143]. Fearful context could preferentially trigger such compensation because threat cues demand rapid disambiguation and typically recruit strong attentional prioritisation. Under this view, enhanced OFG → V1 feedback in fearful context is a state-dependent rescue strategy—but one that may be inefficient or misdirected, explaining why behaviour does not necessarily improve.

(b) Abnormally increased reliance on priors under threat (biased predictive templates)

Recurrent processing is the neural substrate of template-based inference: higher-level interpretations shape lower-level feature sampling. Under threat, priors may become more conservative. If schizophrenia increases reliance on priors when sensory evidence is weak, fear context could amplify this reliance, producing stronger top-down drive into early cortex. This interpretation is compatible with fear being a privileged context through subcortical threat systems (pulvinar–amygdala circuitry) that can bias cortical processing [131,132].

(c) Reduced ability to suppress irrelevant threat context (failure of top-down filtering)

In many attention models, feedback is not merely facilitatory—it can be suppressive, implementing filtering and distractor inhibition. Negative or “inhibitory” effective connectivity in controls under a fearful crowd context could reflect adaptive suppression of distracting peripheral threat cues to protect target processing. In schizophrenia, the reversal toward positive feedback could reflect impaired filtering: threat context continues to invade early visual coding, forcing the system into a heightened, less selective state. The pulvinar’s role in gating and coordinating cortical processing makes it a plausible mediator of this failure mode [131].

(d) Threat-triggered amplification through amygdala–visual interactions (hyper-reactive gain modulation)

An extensive literature links the amygdala to enhanced processing of emotionally salient stimuli in the visual cortex, often conceptualised as increasing the gain of sensory representations. Studies of awareness/visibility and emotional faces show coordinated amygdala–extrastriate effects consistent with amplification when threat cues reach processing thresholds [133,134,135]. If schizophrenia involves dysregulated salience attribution, fear context could lead to an exaggerated gain response, implemented as a stronger top-down drive from higher ventral areas (including occipito-fusiform cortex) to early visual cortex—mainly when the broader system is already operating under reduced sensory precision.

These mechanisms are not speculative; they are consistent with independent evidence that effective connectivity between ventral temporal (including fusiform) regions and occipital cortex is dynamically modulated during perceptual conditions that change the need for recurrent refinement. For example, DCM work in healthy participants has shown that face/body identity processing can modulate backward connections from fusiform regions to occipital cortex, demonstrating that feedback is a controllable physiological mechanism rather than a fixed anatomical detail [149]. Likewise, work linking visibility to effective connectivity between V1 and fusiform cortex supports the broader claim that recurrent coupling is a mechanism of perceptual access and stabilisation [128,129].

#### 4.2.5. Linking Altered Feedback to Alexithymia: From Visual Inference to Emotional Insight

The observed association between fear-specific top-down coupling and alexithymia is theoretically coherent if alexithymia is treated not as a purely “linguistic labelling” problem but as a deficit in constructing or accessing emotion concepts from bodily and perceptual evidence. Contemporary neurobiological accounts of alexithymia emphasise disrupted coordination among affect-generation systems, interoceptive representation (notably insula), and conscious access/report networks (including medial prefrontal and cingulate systems) [150]. Importantly, schizophrenia samples specifically characterised by alexithymia show insula-centred abnormalities during interoceptive tasks, including reduced anterior insula activation and reduced connectivity between anterior insula and ACC/SFG—exactly the circuitry one would expect to integrate salient perceptual cues with internal feeling states and explicit evaluation [151].

How does this relate to recurrent visual feedback? One parsimonious bridge is that unstable or biased perceptual inference about socially relevant threat cues increases downstream uncertainty about one’s own affective state. If fearful context drives excessive or poorly tuned feedback into early visual cortex, the system may generate a percept that is salient but not well calibrated—leading to heightened arousal without precise interpretability. Under this account, stronger fear-specific OFG → V1 coupling would mark a perceptual control strategy that emphasises threat-driven stabilisation of sensory evidence at the expense of accurate, discriminative emotional meaning, thereby aligning with alexithymic difficulty in identifying and describing emotions.

### 4.3. Auditory Processing: When Deficits Emerge Late, and Why They Track Motivation

Across auditory paradigms in schizophrenia, a consistent picture has emerged in which basic registration of sound can be relatively spared under some conditions, while later, integrative stages—those that depend on context formation, salience assignment, and coordinated engagement of fronto–parietal systems—are more reliably disrupted. Your multimodal result in the passive-tone paradigm fits this staging well: the early N100-linked spatiotemporal pattern was not robustly different between groups, whereas the later P200-linked pattern was diminished in patients and—crucially—scaled with avolition/apathy. That combination (late emergence + motivational linkage) is not incidental; it is mechanistically expected if schizophrenia primarily perturbs precision control/gain, network switching, and recurrent integration rather than the earliest feedforward encoding per se.

An extensive EEG literature supports the idea that schizophrenia-related abnormalities strengthen as processing moves from early sensory encoding toward deviance detection and context updating. Mismatch negativity (MMN)—an automatic deviance response that depends on forming a short-term auditory model—shows a robust reduction in schizophrenia across studies and designs [1,152]. This matters for interpretation because MMN is “early” in latency yet already reflects context-dependent inference rather than raw detection, and MMN reductions are commonly framed as impaired prediction-error computation or impaired precision-weighting within auditory hierarchies [153]. Likewise, later oddball-linked components such as P300 show significant, reliable reductions (and latency changes) in schizophrenia across meta-analyses, consistent with impaired allocation of attentional resources and updating of task context [2,7]. P200 itself sits in between; historically underemphasised, it has been shown to be systematically abnormal in schizophrenia, with meta-analytic evidence that the direction of effect depends on whether the stimulus is a standard versus a target—precisely what one would expect if the component is sensitive to stimulus significance and attentional state rather than pure sensory energy [154].

This latency-dependent pattern has a straightforward circuit interpretation. Early auditory ERP components (including N100) are dominated by relatively local thalamo–primary auditory cortex drive and short-range intracortical processing; later components such as P200 and primarily P300 increasingly reflect recurrent loops and long-range coordination: auditory cortex interacting with frontal and parietal control systems, the cingulo-opercular/salience apparatus, and thalamic gating that stabilises the relevant “task set” (even in passive paradigms, a minimal set—stay awake, maintain sensory readiness—must be maintained). When those recurrent loops are inefficient, sensory systems can still respond. Still, responses become less selectively amplified and less functionally coupled to the broader networks that support sustained engagement and context maintenance.

Three converging unimodal lines of evidence reinforce this account.

First, auditory cortical structure and function are altered in ways that bias the system toward unreliable integration rather than absent encoding. Structural MRI reviews and meta-analytic work repeatedly implicate superior temporal regions among the most consistent loci of grey-matter reduction in schizophrenia [22,155]. More recent work links auditory cortical morphology to auditory ERP features, consistent with a structure–function bridge in which reduced cortical thickness or altered microarchitecture constrains the fidelity of population responses [156]. Such alterations are likely to disproportionately affect later, recurrently shaped responses (P200/P300) because recurrent integration relies on intact laminar and inhibitory circuitry to stabilise representations across tens to hundreds of milliseconds.

Second, E/I balance and temporal precision abnormalities in schizophrenia provide a mechanistic route to “late-emerging” deficits. Gamma-band synchronisation during auditory stimulation (e.g., 40 Hz ASSR) is robustly impaired in schizophrenia in meta-analytic work, consistent with disruptions of fast inhibitory–excitatory coordination that supports precise temporal integration [13,157]. These disturbances need not abolish early evoked responses; instead, they reduce the network’s ability to maintain stable phase relationships and to boost signal-to-noise during recurrent processing—exactly the computations that become increasingly important by the P200 window (selection, integration, and early context updating) and beyond (P3-level updating).

Third, context computation/predictive processing accounts make a specific prediction: responses that depend on forming and updating an internal model should be abnormal even when early sensory drive is adequate. MMN work explicitly supports this framing, including evidence consistent with disrupted prediction-error computation in schizophrenia [152,154]. A P200-linked deficit in passive listening can be interpreted within the same framework: it reflects a failure to stabilise a context model (what matters now? what is the precision of this stream?) and to propagate that model through fronto–temporal loops that would usually suppress irrelevant activity (including default-mode engagement) and upweight task-relevant sensory channels.

The multimodal finding that diminished P200-linked expression covaried with greater activity in default-mode midline regions (and relatively greater occipital/visual involvement) is also consistent with this mechanistic story. DMN intrusion during externally driven processing has repeatedly been interpreted as insufficient suppression of internally oriented dynamics and inefficient switching into task-positive modes. Even outside of explicit cognitive tasks, schizophrenia shows altered relationships between intrinsic network dynamics and electrophysiological measures that index large-scale coordination, often implicating DMN hubs [22,158]. The key point for auditory processing is that “late auditory deficits” can be understood less as an auditory-specific failure and more as a failure of global coordination that becomes visible at latencies when coordination is required.

Why, then, should these late auditory abnormalities track motivation/avolition so strongly?

Avolition/apathy is increasingly tied to dysfunction in fronto–striatal valuation and effort-allocation circuitry, not merely to “reduced pleasure.” Contemporary behavioural-neurocomputational work emphasises that schizophrenia involves reduced willingness to expend effort for reward, and that this maps onto altered neural recruitment during effort-based decisions [159]. Meta-analysis of reward processing shows ventral striatal hypoactivation during reward anticipation in psychosis, a plausible neural substrate for diminished energisation and reduced precision assigned to prospective action outcomes [160]. More directly, ventral striatal hypofunction during effort-based decision-making has been shown to reflect dimensional motivational impairment in schizophrenia [161]. These valuation/energisation mechanisms interact tightly with salience and control systems (ACC, anterior insula, dorsolateral prefrontal cortex) that allocate cognitive resources to incoming stimuli.

From that perspective, a reduced P200-linked auditory pattern in individuals with higher avolition is interpretable as a downstream physiological signature of under-allocation of precision/effort to sensory streams. The P200 window is early enough to be “perceptual,” but late enough to reflect whether the system is investing resources into stabilising the sensory model and preparing downstream updating. If motivational circuitry is hypoactive, the organism is less likely to enter (and sustain) a high-engagement mode—even during passive listening—leading to weaker recruitment of fronto–parietal and auditory–attentional coupling, and relatively greater persistence of DMN dynamics. In other words, avolition is not just a behavioural symptom; it plausibly reflects a trait-like reduction in the brain’s propensity to mobilise and maintain task-positive gain states.

This framing also aligns with the broader observation that early sensory-linked measures often predict functional outcomes and real-world capacity more than they predict classical symptom totals. For example, MMN has been repeatedly connected to cognition and functioning, including work linking MMN to domains relevant for independent living and work functioning [162,163]. These associations are conceptually coherent: both MMN and P200/P300 family components index how effectively the brain builds, updates, and uses internal models to guide behaviour over time—capacities that are central to motivated, goal-directed functioning.

Finally, the motivational linkage is also consistent with heterogeneity: schizophrenia is not characterised by a uniform early auditory deficit. Some studies find N100 and gating abnormalities, others find mixed results, and some show that abnormalities are more apparent when the paradigm requires sustained engagement or when gating is explicitly probed [156,164]. If the core deficit concerns gain/precision control and large-scale coordination, then whether an early component appears “impaired” will depend on task demands, arousal, medication, and individual differences in negative symptoms. In contrast, later components, which by design require more coordination, will show more stable group effects and more meaningful links to avolition.

### 4.4. Default Mode Interference, Salience Switching, and “Triple-Network” Dynamics

Across the multimodal findings, a recurrent motif is not simply “too little activation” or “too much connectivity,” but an altered coordination rule among three large-scale systems: the default mode network (DMN), the salience network (SN; anchored in anterior insula and dorsal anterior cingulate), and the central executive network (CEN; frontoparietal control). This “triple-network” framing—articulated initially as a general systems account of cognitive control and psychopathology—provides a valuable bridge between schizophrenia’s phenomenology (intrusive internal mentation, aberrant salience, impaired goal maintenance) and the specific EEG/fMRI signatures observed here [165,166].

Mechanistically, the triple-network model is about state transitions. In healthy cognition, internally oriented DMN modes are down-regulated when external demands rise; SN detects biologically or task-relevant events and helps initiate switching; CEN maintains task sets and supports sustained, effortful processing. A key experimental demonstration of this architecture showed that right anterior insula/fronto–insular cortex (often together with dorsal ACC) behaves like a switch: its activity predicts and helps drive transitions between DMN and executive control systems [166,167]. In schizophrenia, the empirical picture that has emerged over many task and rest paradigms is consistent with a two-part disturbance: (i) DMN interference—reduced task-related DMN suppression and/or excessive coupling of DMN with task systems; and (ii) inefficient salience-mediated switching—abnormal anterior insula/ACC signalling and altered SN coupling to DMN and CEN.

#### 4.4.1. DMN Interference as a Systems-Level Mechanism for Cognitive and Perceptual Inefficiency

A robust fMRI literature indicates that schizophrenia is associated with diminished task-induced deactivation of DMN nodes (especially medial prefrontal and posterior cingulate/praecuneus), most clearly during working memory and attention tasks. This is important because DMN “failure to deactivate” is not merely an imaging curiosity; it is a plausible systems mechanism for subjective intrusions (self-referential mentation, ruminative or associative drift) and objective inefficiency (poorer accuracy, slower reaction times) when externally oriented processing is required [168,169,170].

In schizophrenia specifically, studies using working-memory challenges such as the n-back have reported inefficient DMN suppression, including in early or first-episode samples—suggesting this is not only a chronicity artefact [169,170]. Within the present multimodal results, this DMN-interference interpretation is reinforced by paradigms in which later-stage neural signatures of stimulus processing covary with increased engagement of DMN regions, implying that when task-positive sensory/control networks should dominate, internally oriented networks remain insufficiently down-regulated. This aligns closely with broader accounts that place DMN deactivation deficits at the centre of schizophrenia’s cognitive phenotype [168].

#### 4.4.2. Salience Switching Failure: Anterior Insula/ACC as a Bottleneck for Network Control

If DMN interference is the “symptom-facing” part of the story (intrusions, disengagement, distractibility), the control-side bottleneck is often localised to the SN—particularly anterior insula and dorsal ACC. The SN has been explicitly modelled as the system that flags salient events and initiates control signals that reconfigure large-scale networks [166,167]. In schizophrenia, both functional and structural evidence converge on this circuitry as compromised.

On the functional side, insula-centred abnormalities have been described as altered “between-network connectivity” patterns involving DMN and CEN, in some work even during periods of clinical remission, suggesting a trait-like control vulnerability [171]. On the structural side, meta-analytic evidence indicates reductions in insular grey matter in schizophrenia, with clinical relevance to hallucinations and broader dysfunction—consistent with the idea that compromised salience hubs limit efficient state switching [172]. More recent work continues to identify salience-network segregation and altered community structure as relevant to symptom profiles, including negative symptom dimensions [173].

A particularly mechanistic way to interpret these convergences is to treat anterior insula/ACC dysfunction as producing a lower signal-to-noise “switch command”. When the switch signal is weak or mistimed, DMN remains partially engaged during tasks, and executive control is recruited inefficiently or compensatorily. This naturally predicts the kinds of observations recurring across the summarised multimodal studies: behavioural performance may be preserved in easier conditions (because compensatory recruitment is possible), yet neural signatures reveal inefficient allocation (DMN persistence, altered coupling, delayed or frequency-shifted relationships between electrophysiology and BOLD).

#### 4.4.3. Oscillatory and Subsecond Signatures: Why EEG Often Shows “Frequency Shifts” and Altered Microstate Dynamics

A significant advantage of combining EEG reasoning with network models is that switching is intrinsically a temporal process. fMRI captures the network topology and slower state occupancy; EEG can index the fast control signals and gating rhythms that plausibly implement switching.

In healthy samples, trial-by-trial EEG–BOLD work has linked alpha and theta fluctuations to distinct distributed networks relevant to attention and working memory, supporting the view that oscillations help coordinate large-scale systems rather than merely reflecting local “idling” [174,175]. Alpha-band synchronisation has also been linked to frontoparietal control and alertness regulation, consistent with a mechanistic role in stabilising task sets and suppressing irrelevant processing streams [176].

Two EEG studies are especially informative for the present findings.

The first are EEG microstates as sub-second network switching units. Microstates are quasi-stable scalp topographies lasting on the order of ~50–150 ms, and they have been proposed as electrophysiological correlates of large-scale resting-state networks [177,178]. In schizophrenia, a meta-analysis spanning many studies reported systematic microstate abnormalities (e.g., altered prevalence and duration of specific canonical microstate classes), suggesting a trait-level disturbance in the rapid sequencing of global brain states [179]. Work in prodromal/high-risk samples also reports microstate deviations, implying that altered microstate dynamics may index vulnerability in the same control architecture that later manifests as impaired salience switching and DMN interference [180]. More recent large-scale datasets have strengthened the endophenotype case by showing microstate alterations across psychosis spectrum groups with genetic and clinical relevance [181].

The second are “Frequency downshifts” and altered cross-network coupling. Several EEG–fMRI literatures in healthy subjects show that alpha power and related metrics are tied to anticorrelations between DMN and attention systems, and that changes in oscillatory regime can reweight the balance between internally and externally oriented processing [174,175]. Against that backdrop, schizophrenia-associated “frequency shifts” (where network-linked signatures appear at slower rhythms) can be interpreted as a control-system slowing; switching signals become less phasic and more sluggish, forcing task systems to operate with reduced temporal precision. This is a plausible physiological route to the empirical pattern seen across many schizophrenia EEG studies (increases in slower rhythms and alterations in higher-frequency coordination), and it matches the broader idea of dysregulated gain and excitation–inhibition balance as a substrate for unstable or mistimed network control [165,182].

#### 4.4.4. Neuromodulatory Control and Salience: Dopamine, Thalamus, and “Precision” of Switching

Finally, triple-network dynamics should be situated within neuromodulatory and subcortical control systems. Salience assignment and switching plausibly depend on dopaminergic signalling because dopamine is centrally implicated in learning what matters (precision weighting, motivational vigour, and the attribution of salience). The aberrant salience framework and its dopamine imaging extensions provide a mechanistic bridge from striatal dysregulation to cortical network instability; if salience signals are noisy or miscalibrated, the SN’s gating function becomes unreliable, and DMN/CEN transitions become either excessive (distractible switching) or insufficient (perseveration, disengagement) [165,182,183]. PET meta-analytic work supports altered presynaptic dopamine function in schizophrenia (with heterogeneity across patients), which is compatible with variable degrees and forms of switching impairment and with symptom-linked subtypes [184,185].

In addition, thalamic and thalamo–cortical circuitry are well positioned to modulate large-scale network interactions (broadcasting, filtering, and synchronising cortical activity). While the present multimodal results emphasise rhythm-dependent thalamus–network coupling abnormalities, this fits a broader mechanistic view in which thalamic gating and cortical oscillations jointly implement the “routing” operations required for stable DMN suppression and flexible engagement of executive systems.

### 4.5. Thalamo–Cortical Filtering and Hierarchical Organisation

A convergent theme across the present multimodal findings is that moment-to-moment coordination—not mean activity—appears altered; rhythm-dependent thalamo–cortical and network interactions become less specific, and large-scale systems show timing and hierarchy changes. A parsimonious mechanistic interpretation is that schizophrenia involves a disturbance of thalamo–cortical “filtering” (precision control over what signals are amplified vs. suppressed) that then propagates upward to the cortex-wide functional hierarchy, altering how sensory evidence, salience, and internally generated activity are integrated over time.

#### 4.5.1. Thalamus as a Precision Filter: TRN-Mediated Inhibition, Rhythmic Gating, and Corticothalamic Control

The thalamus is not a passive relay. Its communication with the cortex is continuously sculpted by inhibitory control from the thalamic reticular nucleus (TRN) and by reciprocal corticothalamic feedback. The TRN is strategically positioned to regulate thalamic throughput via GABAergic inhibition, shaping both sensory selection during wakefulness and sleep spindle generation during NREM sleep—processes that are tightly linked to attention, learning, and stabilisation of cortical representations. Reviews specifically framing TRN dysfunction as central to schizophrenia emphasise that TRN–cortex–thalamus loops simultaneously support bottom-up filtering and top-down control, and that perturbations can plausibly yield both sensory flooding and higher-order disorganisation [186,187].

Oscillations provide the operational format for this filtering. Spindles, in particular, are generated by TRN–thalamic interactions and depend on intact corticothalamic modulation; they are therefore an in vivo signature of the integrity of thalamo–cortical inhibitory timing. Extensive empirical literatures show robust reductions in spindle activity in schizophrenia, consistent with impaired TRN-linked inhibitory coordination and reduced capacity to “stabilise” cortical network states [188,189,190]. Complementing this, animal-model work demonstrates that disruption of TRN parvalbumin neuron function can produce abnormalities in cortical delta/gamma/spindle activity, providing a mechanistic bridge between cellular inhibitory deficits and systems-level dysrhythmia [191].

Within this framework, the thalamus becomes a precision controller; it modulates the gain of ascending sensory evidence and helps align cortical excitability to behaviourally relevant rhythms. When TRN-mediated inhibition or corticothalamic calibration is weakened or mistimed, thalamo–cortical signalling can become both noisier and less selectively modulated by context—precisely the kind of “loss of specificity” that would appear as reduced rhythm selectivity or altered timing in EEG–BOLD relationships without necessarily changing mean EEG power.

#### 4.5.2. Sensory Gating and “Failed Suppression”: Linking Thalamus, Hippocampus, and Prefrontal Cortex

A classic behavioural-physiological correlate of impaired filtering in schizophrenia is diminished sensory gating (e.g., reduced suppression of responses to repeated stimuli). fMRI studies of gating paradigms have repeatedly implicated a distributed hippocampus–thalamus–prefrontal network, consistent with the idea that gating failures reflect abnormal inhibitory regulation of thalamo–cortical transmission and its contextual control. For example, Tregellas and colleagues reported abnormal hemodynamic engagement of the hippocampus, thalamus, and prefrontal cortex during impaired gating in schizophrenia. They interpreted this pattern as consistent with diminished inhibitory function in the gating circuit [192,193].

Mechanistically, hippocampal dysregulation can amplify thalamic and cortical responsiveness by biasing salience/novelty processing, while prefrontal dyscontrol weakens top-down constraints that typically stabilise gating thresholds. The resulting state is not simply “more activation.” Still, in a reconfiguration of coupling, (i) thalamic channels become less suppressible, (ii) cortical networks show reduced ability to enter task-appropriate low-entropy states, and (iii) oscillatory markers of gating (alpha/spindle-range rhythms, and their cross-frequency organisation) become unreliable.

#### 4.5.3. Thalamo–Cortical Dysconnectivity: Sensory Hyperconnectivity, Prefrontal Hypoconnectivity, and Symptom Relevance

Large-scale resting-state fMRI evidence strongly supports the thalamus as a hub of schizophrenia-associated dysconnectivity. A major multi-site brain-wide association study (hundreds of patients and controls) identified the thalamus as the key locus of altered functional connectivity, with a reproducible pattern: increased thalamic connectivity with primary sensory/motor cortices and reduced thalamo–frontal connectivity [194]. This “sensory up/frontal down” motif provides a systems-level explanation for why patients can show both heightened sensory interference (bottom-up capture, reduced filtering) and weakened executive stabilisation (top-down control).

Meta-analytic syntheses and focused studies likewise highlight thalamo–cortical network alterations as among the most robust resting-state findings in schizophrenia, with particular involvement of mediodorsal and pulvinar-related circuits that link thalamus to prefrontal and parietal control systems [195,196]. Importantly, thalamic abnormalities are not purely functional; contemporary structural work resolving thalamic nuclei indicates that schizophrenia is associated with nucleus-specific volume alterations (with different nuclei showing different directions and magnitudes of change), consistent with circuit-selective disruption rather than a uniform thalamic “shrinkage” account [197].

#### 4.5.4. From Thalamic Filtering to Cortex-Wide Hierarchy: Gradients, Segregation, and Temporal Organisation

The functional hierarchy of the cortex can be summarised as a smooth axis (or set of axes) running from unimodal sensory regions to transmodal association cortex; disruptions of this hierarchy offer a modern formalisation of “fronto–occipital disconnectivity.” Resting-state fMRI gradient and hierarchy approaches in schizophrenia increasingly report that hierarchical organisation is altered in ways consistent with reduced differentiation between sensory and transmodal systems, or abnormal re-weighting of the hierarchy at the whole-brain and network levels.

For example, a *Psychological Medicine* study of drug-naïve first-episode schizophrenia reported functional gradient dysfunction. It linked these hierarchical alterations to biological features and treatment-related predictions, supporting the view that hierarchy disruption is present early and is not simply a chronicity/medication artefact [198]. More recent work using alternative hierarchy formalisms (e.g., quantifying deviations from equilibrium in effective connectivity models) reports that hierarchical organisation is measurably reconfigured in schizophrenia and relates to clinical features, including negative-symptom adjacent dimensions such as apathy [199].

Mechanistically, thalamic filtering is a plausible driver of hierarchy change because thalamo–cortical pathways set the reliability (precision) of sensory evidence and influence which cortical levels dominate competition for representation. If thalamic gating is weakened, sensory systems may exert disproportionate, noisy influence (“sensory hyperweighting”), while association cortex fails to impose stable top-down constraints (“control underweighting”). Over time, this should manifest as altered hierarchical segregation: weaker separation between sensory and transmodal systems, abnormal coupling between default-mode regions and perceptual streams, and less consistent oscillatory-temporal alignment between neural events and slower hemodynamic organisation.

In the present multimodal pattern, hierarchy change is therefore interpretable as the systems-level footprint of thalamo–cortical precision dysregulation. The most important implication is that a single abnormal network may not characterise schizophrenia. Still, by an altered operating regime: (i) thalamic “gates” are less selective, (ii) cortical hierarchy becomes harder to stabilise, and (iii) network transitions become less reliably orchestrated by salience/control systems—yielding the observed coexistence of sensory interference, default-mode intrusion, and reduced context sensitivity across tasks and rest.

### 4.6. Timing Matters: Noncanonical EEG–BOLD Relationships as a Physiological Mechanism

A central interpretive risk in multimodal electrophysiology–fMRI work is to treat the EEG → BOLD relationship as if it were governed by a single “canonical” hemodynamic response function (HRF); neural activity changes first, and a few seconds later, BOLD follows with a stereotyped rise and fall. In practice, the latency, dispersion, and even polarity of BOLD responses vary substantially across brain regions and across individuals, even in healthy participants. Foundational work showed that HRF timing and shape differences can be significant enough to bias effect estimates and group comparisons if a single fixed HRF is assumed [200,201]. This variability is not just measurement noise; it reflects absolute heterogeneity in vascular anatomy, baseline perfusion, vascular reactivity, and neurovascular coupling mechanisms across cortex and subcortex [202,203,204].

Against that background, “noncanonical” EEG–BOLD timing—where the strongest coupling occurs at unusually short lags (near 0–2 s), at longer-than-expected lags, or even with apparent lead–lag reversals—should not be treated as a nuisance artefact by default. Instead, it can be a mechanistically informative signature of which physiological process is dominating the coupling at that moment: local neurovascular transduction, large-vessel/vascular propagation, systemic physiology (respiration/CO_2_), or slow brain-state dynamics (arousal) that jointly modulate neuronal synchronisation and blood flow.

#### 4.6.1. Noncanonical Timing as an Emergent Property of Resting-State BOLD Dynamics

Resting-state BOLD is not temporally homogeneous across the brain; multiple groups have shown that spontaneous BOLD fluctuations exhibit a reproducible lag structure—a spatiotemporal organisation in which signals appear to propagate across networks with regionally specific delays [205]. Importantly, these lags can arise from at least two partially overlapping phenomena. One is hemodynamic/vascular propagation; low-frequency oscillations in blood flow and oxygenation can travel along vascular pathways, producing time-lagged correlations that reflect circulation rather than synaptic signalling. Another is neurally organised propagation; structured transitions among network states can generate apparent delays even when the underlying neural events are relatively synchronous at the timescale of seconds. The key point for EEG–BOLD coupling is that both mechanisms predict regional differences in the timing of BOLD fluctuations. Still, they imply different interpretations of “early” versus “late” coupling; early coupling may indicate either fast local transduction in certain territories or dominance of a non-neural vascular component that is temporally aligned across widespread areas.

A major challenge in interpreting the current multimodal EEG–MRI literature is the substantial methodological heterogeneity across studies. Included investigations differed considerably in EEG preprocessing pipelines, artefact-removal strategies, scanner parameters, acquisition timing (simultaneous versus separate-session EEG/fMRI), resting-state conditions, task paradigms, and multimodal fusion methodologies. Variability in gradient and ballistocardiographic artefact correction, ICA/CCA implementation, EEG referencing schemes, and feature extraction procedures may substantially influence electrophysiological measures and cross-modal coupling estimates. Similarly, differences in MRI acquisition parameters, motion correction procedures, network definitions, and statistical thresholds complicate direct comparison across datasets. Fusion approaches themselves (e.g., EEG-informed fMRI, jICA, mCCA, ACMTF, DCM-based integration) rely on distinct mathematical assumptions and may capture partially different aspects of multimodal brain organisation. Consequently, methodological variability likely contributes substantially to between-study inconsistencies and currently limits reproducibility and cross-study generalisability. Future progress will likely depend on greater harmonisation of acquisition protocols, preprocessing standards, and multimodal integration frameworks across research sites.

#### 4.6.2. Clinical and Translational Implications

The reviewed literature also highlights the potential clinical and translational relevance of multimodal EEG–MRI approaches in schizophrenia research. Several studies demonstrated that abnormalities in EEG–BOLD coupling, joint multimodal components, and large-scale network integration may be detectable even when unimodal EEG or MRI findings are weak, inconsistent, or nonspecific. This suggests that multimodal approaches may provide greater sensitivity for identifying clinically meaningful circuit dysfunction than either modality alone.

Particularly important are findings from clinical high-risk and early psychosis cohorts, in which altered gamma-related coupling and network abnormalities were observed despite limited conventional fMRI group differences. Such observations raise the possibility that multimodal EEG–MRI biomarkers could eventually contribute to earlier detection of psychosis vulnerability and improved stratification of individuals at elevated clinical risk. Similarly, multimodal approaches combining electrophysiological and hemodynamic information may help characterise symptom-relevant network disturbances associated with cognitive dysfunction, salience processing abnormalities, auditory prediction deficits, and impaired executive control.

Several studies also suggested that multimodal integration methods, including joint ICA, mCCA, ACMTF, and EEG-informed fMRI approaches, may improve classification performance and reveal biologically meaningful patterns that are not apparent in unimodal analyses alone. In principle, this could support future precision psychiatry frameworks aimed at identifying biologically distinct subgroups characterised by differing patterns of dysconnectivity, oscillatory dysfunction, or abnormal network organisation. Such approaches may eventually assist in predicting symptom trajectories, cognitive outcomes, or treatment response.

However, despite these promising developments, current evidence remains insufficient for routine clinical implementation. Many studies remain limited by small sample sizes, medication heterogeneity, demographic imbalance, methodological variability, and lack of longitudinal replication. In addition, substantial differences in preprocessing pipelines, multimodal fusion strategies, and statistical procedures continue to limit reproducibility and cross-study comparability. Consequently, multimodal EEG–MRI biomarkers should currently be regarded as promising research tools rather than validated clinical biomarkers. Future progress will likely depend on larger multicentre datasets, longitudinal designs, harmonised acquisition and preprocessing standards, and replication across independent cohorts.

#### 4.6.3. Limitations and Future Directions

A major limitation across the reviewed literature concerns antipsychotic medication exposure. Most included schizophrenia cohorts consisted predominantly of chronically medicated patients, often receiving atypical antipsychotics. Antipsychotic drugs can substantially influence electrophysiological oscillations, spectral power, cerebral blood flow, resting-state connectivity, and neurovascular coupling, thereby potentially altering the very EEG–MRI relationships being measured. Consequently, some reported multimodal abnormalities may partly reflect treatment-related neurophysiological adaptations rather than primary disease mechanisms. Although several studies reported chlorpromazine- or risperidone-equivalent doses and examined medication correlations, pharmacological confounding remains difficult to eliminate in current multimodal schizophrenia research. The restriction to English-language publications may also have contributed to language bias and reduced representation of findings published in non-English regional journals.

Several important limitations currently affect reproducibility and generalisability in multimodal EEG–MRI schizophrenia research. First, many included studies were characterised by relatively small sample sizes, often involving fewer than 30 schizophrenia participants, increasing susceptibility to statistical instability, overfitting, and reduced reproducibility. Small cohorts are particularly problematic in multimodal fusion analyses, where high-dimensional feature spaces may exceed the effective statistical power of available datasets.

Second, substantial clinical heterogeneity was present across studies, including variation in illness duration, symptom severity, diagnostic composition, smoking status, and medication exposure. Most patient cohorts consisted predominantly of chronically medicated individuals receiving antipsychotic treatment, which may influence electrophysiological oscillations, cerebral blood flow, resting-state connectivity, and neurovascular coupling. As a result, distinguishing primary disease-related abnormalities from treatment-related effects remains challenging.

Third, demographic imbalance and sampling variability may further limit generalisability. Several cohorts were strongly male-predominant, restricted to right-handed individuals, or derived from relatively narrow age ranges and geographically localised recruitment samples. In addition, many studies excluded participants with excessive motion or poor EEG quality, potentially introducing selection bias toward higher-functioning or more compliant patient groups.

Methodological heterogeneity also remains a major obstacle to reproducibility. Studies differed substantially in EEG preprocessing pipelines, artefact-removal procedures, MRI acquisition parameters, task paradigms, network definitions, and multimodal integration strategies. Because multimodal EEG–MRI measures are highly sensitive to preprocessing and modelling choices, such variability complicates direct comparison across studies and may contribute to inconsistent findings.

Finally, relatively few studies employed longitudinal designs, first-episode cohorts, minimally medicated samples, or multicentre replication frameworks. Consequently, it remains difficult to determine the temporal stability, illness-stage specificity, and clinical robustness of proposed multimodal biomarkers. Future progress in the field will likely require larger harmonised multicentre datasets, standardised preprocessing frameworks, longitudinal follow-up studies, and greater integration of mechanistically interpretable multimodal models with clinically meaningful outcomes.

## 5. Conclusions

Across 23 eligible studies integrating EEG with MRI modalities (predominantly simultaneous EEG–fMRI, with some sequential EEG+fMRI and a smaller number adding sMRI and/or DTI), the multimodal EEG–MRI schizophrenia literature supports a clear overarching conclusion: the most reproducible disease signals are not simply “smaller ERPs” or “weaker BOLD activation,” but abnormalities in the linkage rules between fast electrophysiological dynamics and spatially distributed brain networks.

First, in domains often framed as “early sensory deficits,” multimodal evidence suggests that schizophrenia-related abnormalities are frequently expressed more reliably in network embedding and top-down regulation than in the earliest evoked peaks alone. Early ERP effects (e.g., N100/MMN-range measures) could be subtle or inconsistent in some datasets. In contrast, multimodal modelling revealed disturbances such as context-dependent feedback to early sensory cortex, later perceptual–attentional components (e.g., P200-linked joint patterns), and structure–function linkages that implicate temporal, thalamic/hippocampal, and frontoparietal circuitry even when scalp metrics are equivocal.

Second, the strongest convergence across tasks appeared in cognitive control, salience, and target detection (especially oddball paradigms). Here, multimodal integration repeatedly sharpened classic findings (P300 reductions, altered task activation) into a more mechanistic statement; patients show reduced expression and/or disrupted coupling between late target-processing electrophysiology (P3/P300; sometimes N2) and recruitment of salience/ventral attention and fronto–temporal control networks (including ACC/anterior insula/TPJ and related circuitry). This “uncoupling” framing was often more informative than either modality alone.

Third, simultaneous EEG–fMRI studies of working memory indicate that psychosis involves not only impaired performance or altered oscillatory scaling, but a reorganisation of state-dependent relationships; preparatory (prestimulus) fMRI network states (notably DMN and attention networks) predict retention-period oscillations in controls, while these dependencies are altered in schizophrenia-spectrum participants. This supports a model in which WM impairment partly reflects disrupted preparation-to-maintenance coupling, rather than a single localised deficit.

Fourth, resting-state studies converge on schizophrenia as a disorder of abnormal EEG–BOLD coupling architecture, expressed through frequency specificity, directionality, and timing differences. Notably, some of the most compelling results emerged precisely when unimodal averages were null (e.g., altered coupling despite similar mean power), reinforcing that multimodal approaches can detect “hidden” physiology—how rhythms, synchronisation, aperiodic components, or traveling-wave directionality map onto DMN hubs, sensory cortex, thalamo–cortical interactions, and large-scale hierarchical organisation.

Fifth, when studies expanded to tri-modal designs (EEG+fMRI+sMRI, and occasionally DTI) and/or classification, adding modalities often improved separability and interpretability. Still, the review also highlights that reported performance is sensitive to sample size, demographic imbalance, and validation/feature-selection choices. Consequently, the most clinically meaningful direction is not “classification at any cost,” but models that preserve mechanistic coupling (structure–function relationships, rhythm-dependent modulation, hierarchy–directionality links) and can be replicated across sites.

Finally, regarding clinical relevance, the reviewed studies suggest that symptoms and phenotypes relate more consistently to multimodal coupling parameters (effective connectivity, joint component expression, coupling strength/timing) than to unimodal amplitudes or mean band power. Medication was frequently considered and often did not straightforwardly account for core coupling abnormalities. However, targeted pharmacologic manipulation (e.g., nicotine) demonstrated that EEG-informed fMRI can be a sensitive readout of neuromodulatory effects on control circuitry.

Multimodal EEG–MRI work supports schizophrenia as a disorder of distributed, multi-scale dysconnectivity in which abnormalities are most clearly revealed by measuring how neural dynamics (EEG) are coupled to network-level organisation and hemodynamic expression (MRI). The field’s next gains will likely come from (i) larger and more diverse samples (including early/CHR and longitudinal designs), (ii) harmonised acquisition and artefact-correction standards for simultaneous EEG–fMRI, and (iii) preregistered, cross-site replication of mechanistic multimodal markers that explicitly model coupling, timing, and directionality—moving from descriptive abnormalities toward explainable, clinically anchored neurobiological assays.

## Figures and Tables

**Table 1:** Studies included in the review.

Study	Sample	Paradigm/Task	Modalities & Analysis	Main Findings
[96]	14 schizophrenia (SZ), 14 healthy controls (HCs)	Emotional face-context task: peripheral crowd faces (fearful/happy/neutral/scrambled) preceding central target face	Simultaneous EEG–fMRI; ERP (P1, N170); fMRI GLM; fMRI-informed EEG–DCM	No clear behavioural context effect, but SZ showed fear-specific abnormal top-down feedback from left occipital fusiform gyrus to V1. Stronger abnormal backward connectivity correlated with alexithymia in SZ.
[97]	48 SZ, 53 HCs	Resting state	rs-fMRI (ALFF) + resting EEG spectra + sMRI GM; 3-way MCCA; SVM classification	Multimodal fusion outperformed single modalities. EEG+GM+ALFF gave the best discrimination, with reported classification around ~90–100% depending on pipeline/testing scheme.
[98]	30 schizophrenia-spectrum, 23 HCs	Passive auditory tone listening	Simultaneous EEG–fMRI; joint ICA (jICA)	Early N100-linked auditory pattern was not significantly different, but P200-linked joint component was reduced in patients. Smaller P200-linked pattern related to greater avolition/apathy.
[99]	11 SZ, 21 HCs	Auditory oddball	EEG tensor + fMRI, optional sMRI; ACMTF/coupled matrix–tensor factorization; comparison with jICA	Fusion yielded interpretable components differentiating groups. EEG–fMRI ACMTF improved clustering accuracy (up to ~88–91%); adding sMRI plus subject-centring also reached ~91%.
[100]	84 SZ, 47 HCs initially; 9 excluded for motion	Resting state, eyes closed	Simultaneous EEG–fMRI; EEG dynamic rhythm info via ADTF; fMRI PPI; network coupling analyses	Schizophrenia altered rhythm-dependent thalamo–cortical and triple-network connectivity (DMN/SN/CEN). Some connections lost rhythm specificity; coupling abnormalities related to illness duration, age of onset, and partly medication.
[101]	57 schizophrenia-spectrum, 46 HCs	Resting state in scanner (eyes open)	Simultaneous EEG–fMRI; EEG-informed fMRI using gamma power and aperiodic exponent	Mean EEG measures did not differ, but SZ showed reduced gamma–BOLD coupling in bilateral superior temporal gyrus/auditory cortex and altered exponent–BOLD coupling in superior frontal/SMA. Weaker right STG coupling related to worse sensory gating and higher symptoms.
[102]	12 SZ, 15 HCs	Auditory omission MMN paradigm	Simultaneous EEG–fMRI + DTI	No significant MMN amplitude/latency group difference after covariates, but SZ showed reduced omission-related temporal lobe BOLD (especially MTG). White-matter abnormalities involved ACC-related tracts; some structure–function and symptom associations were reported.
[103]	16 SZ, 22 HCs	Auditory oddball	EEG tensor + fMRI; EEG-only CP/PARAFAC; fused ACMTF/CMTF	EEG-only components already separated groups, but fusion produced linked spatiotemporal components. The 11-electrode subset yielded especially clear results, linking P3/N2–P3 features to DMN, superior parietal, and visual fMRI patterns.
[104]	~107 SZ, 57 relatives, ~108 controls (usable N varied by paradigm)	rsEEG, MMN, 40 Hz ASSR, rsfMRI	Multimodal DCM/PEB across EEG and fMRI	Across modalities, findings converged on a reduced synaptic gain/increased self-inhibition account, especially in frontal pyramidal populations. Symptom-linked patterns suggested additional circuit disinhibition, particularly for auditory abnormalities.
[105]	17 psychotic-spectrum patients, 17 HCs	Verbal Sternberg working memory task	Simultaneous EEG–fMRI; prestimulus fMRI network states linked to retention-period EEG oscillations	Patients were slower and less accurate at high load. Controls showed expected preparatory DMN suppression/dAN engagement and a normal inverse prestimulus DMN–frontal theta relation; this coupling was altered/disrupted in patients.
[106]	24 high-risk for psychosis (HRP), 24 HCs	Auditory choice-reaction task	Simultaneous EEG–fMRI; EEG-informed fMRI using single-trial auditory evoked gamma-band response (aeGBR)	HRP showed reduced aeGBR power and reduced BOLD responses associated with trial-wise gamma-band fluctuations in a fronto–temporal/thalamic/ACC/DLPFC network, despite no corrected conventional fMRI group differences.
[107]	42 psychotic patients, 37 HCs across two sites	Resting state	Simultaneous EEG–fMRI; EEG Global Field Synchronization (GFS); EEG-informed fMRI	Psychosis was associated with reversed or abnormal GFS–BOLD coupling in DMN regions (especially delta and beta bands) and altered alpha1 coupling in extrastriate visual cortex. Effects were not explained by EEG power alone.
[108]	99 SZ, 56 HCs final sample	Resting state	Simultaneous EEG–fMRI; fMRI functional gradients/eccentricity + EEG traveling waves	SZ showed reduced fronto–occipital hierarchical separation and increased theta forward-wave power. Coupling between cortical hierarchy and wave directionality was reorganized: stronger frontal/weaker occipital relationships, with clinical moderation by symptoms and illness duration.
[109]	11 schizophrenia-spectrum patients, 11 HCs	Resting state, eyes closed	Simultaneous EEG–fMRI; ICA-derived DMN and left working-memory/language network; EEG–RSN covariance mapping	Patients showed altered RSN connectivity and a frequency “downshift” in EEG–RSN coupling: coupling signatures that occurred at higher frequencies in controls appeared at lower frequencies in patients, especially for DMN and LWMN.
[111]	23 male SZ, 20 male HCs initially; smaller analysable subsamples for fMRI/P50	Somatosensory sensory gating (median nerve paired stimulation)	Simultaneous EEG–fMRI	Controls showed significant P50-like gating in the 500 ms condition; patients did not. Better gating related to stronger single-stimulus BOLD in hippocampus, thalamus, STG, and left IFG. Patients also showed reduced activation in some gating-related regions.
[111]	14 schizophrenia smokers, 15 healthy smokers	Visual oddball after nicotine vs. placebo nasal spray	Simultaneous EEG–fMRI; EEG-informed fMRI using single-trial P300 amplitudes	Nicotine did not clearly change behaviour or average P300 amplitude, but EEG-informed fMRI detected increased ACC/medial frontal activation under nicotine in both groups. Conventional fMRI was less sensitive.
[112]	22 SZ, 22 HCs	Auditory oddball	Simultaneous EEG–fMRI; single-trial P300 and oscillatory regressors; ICA/network analyses	Patients showed reduced P300, delta, theta, and alpha responses. In controls, P300 correlated with superior parietal/praecuneus activation; this linkage was absent in patients, indicating disrupted brain–behaviour coupling.
[113]	14 SZ, 15 HCs	Resting state, eyes closed	Simultaneous EEG–fMRI; rsfMRI CONN seed/ROI analyses + EEG source-space lagged coherence	No robust within-DMN group differences in either modality. Main effect was increased mPFC–right posterior inferior temporal gyrus connectivity in SZ on fMRI ROI-to-ROI analysis; cross-modality correspondence was generally weak.
[114]	16 chronic SZ, 23 HCs	Auditory oddball	Separate ERP + fMRI sessions; joint ICA fusion	One joint component significantly differentiated groups, linking an N2-range ERP abnormality with reduced bilateral fronto–temporal fMRI activation. Supports a coupled electrophysiological–hemodynamic deficit during target processing.
[115]	57 schizophrenia patients, 46 unaffected participants	Resting state, eyes open	Simultaneous EEG–fMRI; lagged correlation of residual alpha power with BOLD	Resting EEG–BOLD coupling showed noncanonical timing, especially in DMN. SZ showed more delayed/right-shifted coupling in several cortical/subcortical regions, including praecuneus, fusiform, superior frontal, and pregenual ACC.
[116]	21 SZ, 22 HCs	Visual oddball	Separate ERP and fMRI sessions; jICA	Early sensory P100/N100 processing was relatively preserved, but patients had reduced P300 and reduced expression of a joint P300–ventral attention/salience network component involving ACC, anterior insula, and TPJ.
[117]	24 SZ, 25 HCs final sample	Picture–word semantic mismatch task	Separate ERP (N400) + fMRI sessions; joint ICA	SZ showed reduced loading on a joint N400–fMRI component. The hemodynamic network linked to N400 differed by semantic relation type, and reduced N400-linked network activity was associated with unusual thought content.
[118]	Dataset 1: 16 SZ, 23 HCs (fMRI+EEG oddball); Dataset 2: 37 SZ, 36 HCs (fMRI+sMRI sensorimotor)	Auditory oddball and sensorimotor task	mCCA multimodal fusion; compared with jICA	mCCA recovered cross-modal relationships more flexibly than jICA. In the oddball dataset, linked temporal/ACC fMRI and N2/P3-related ERP components differentiated groups. In the fMRI+sMRI dataset, coupled functional motor/temporal and frontal/temporal GM patterns separated SZ from HC.

**Table 2:** Risk of bias assessment.

Bias in Selection of the Reported Result	Bias in Measurement of Outcomes	Bias Due to Missing Data	Bias Due to Deviations from Intended Interventions	Bias in Classification of Exposure/Group	Bias in Selection of Participants	Bias Due to Confounding	Study
Moderate to Serious	Moderate	Moderate	Low	Low	Moderate	Serious	[96]
Serious	Serious	Moderate	Low (limited applicability)	Moderate	Moderate to Serious	Serious	[97]
Moderate	Moderate	Serious	Low	Low	Moderate	Serious	[98]
Serious	Serious	Moderate	Low	Low	Moderate	Serious	[99]
Serious	Moderate to Serious	Moderate	Low	Low	Moderate	Serious	[100]
Moderate	Moderate	Low	Low to Moderate	Low	Moderate	Serious	[101]
Serious	Low to Moderate	Moderate	Low to Moderate	Low	Serious	Serious	[102]
Serious	Moderate	No information (cannot judge)	Moderate	Moderate	Serious	Serious	[103]
Moderate	Low to Moderate	Moderate	Low	Moderate	Moderate	Serious	[104]
Moderate	Moderate	Moderate	Low to Moderate	Low	Serious	Serious	[105]
Moderate to Serious	Moderate	Low to Moderate	Low	Low to Moderate	Moderate	Serious	[106]
Moderate to Serious	Moderate	Moderate to Serious	Low to Moderate risk (partly applicable)	Low to Moderate	Moderate	Serious	[107]
Moderate	Moderate	Moderate	Low (largely not applicable)	Low	Moderate	Serious	[108]
Serious	Serious	Moderate	Moderate	Low	Moderate to Serious	Serious	[109]
Serious	Moderate	Serious	Moderate	Low	Serious	Serious	[110]
Moderate	Moderate	Moderate	Low to Moderate	Low	Low	Moderate	[111]
Serious	Moderate to Serious	Serious	Serious	Moderate	Serious	Serious	[112]
Moderate	Moderate	Moderate	Low	Moderate	Moderate	Serious	[113]
Moderate	Moderate	Moderate to Serious	Moderate	Low	Moderate	Serious	[114]
Moderate	Moderate	Moderate	Low	Low	Moderate	Serious	[115]
Moderate	Low to Moderate	Low to Moderate	Low to Moderate	Low	Moderate	Serious	[116]
Moderate to Serious	Moderate	Moderate	Low	Low	Moderate	Serious	[117]
Likely serious	Likely moderate	Likely unclear to moderate	Low (mostly not applicable)	Low to Moderate	Moderate	Serious	[118]

## Data Availability

No new data were created or analysed in this study. Data sharing is not applicable to this article.

## Supplementary Materials

The following supporting information can be downloaded at https://www.mdpi.com/article/10.3390/jcm15114306/s1, Supplementary Table S1: PRISMA 2020 Checklist.
